# Intelligent Recognition of Sulfur-Containing VOCs Using a Au/Pd-Modified RGO-SnO_2_ Gas Sensor Array

**DOI:** 10.3390/ma19214606

**Published:** 2026-10-30

**Authors:** Heng Fu, Lin Shi, Yongfeng Kang, Yukang Liao, Hui Li, Le Zheng, Jian Song

**Affiliations:** 1 School of Microelectronics, Shanghai University, Shanghai 200444, China; 2 State Key Laboratory of High Performance Ceramics, Shanghai Institute of Ceramics, Chinese Academy of Sciences, Shanghai 200050, China

**Keywords:** sulfur-containing VOCs, rGO-SnO_2_, noble metal modification, gas sensor array, ResNet, intelligent recognition

## Abstract

To address the challenge of accurately distinguishing structurally similar sulfur-containing volatile organic compounds (VOCs) using a single gas sensor because of their similar response characteristics, this study developed a gas sensor array based on Au/Pd-modified rGO/SnO_2_ devices for the detection and recognition of 1-butanethiol (BT), dimethyl sulfide (DMS), and allyl methyl sulfide (AMS). rGO and SnO_2_ sensing layers were sequentially fabricated on Si/SiO_2_ substrates, followed by the deposition of Au or Pd and subsequent annealing, resulting in three sensor types: pristine rGO/SnO_2_ Au-modified rGO/SnO_2_, and Pd-modified rGO/SnO_2_. SEM, Raman, and XPS characterizations revealed the nanoscale dispersion of Au and Pd on the rGO/SnO_2_ surface while confirming the carbon framework of rGO and the characteristic vibrational and chemical-state features of SnO_2_. Gas-sensing measurements showed that all three sensor types exhibited clear responses to BT, DMS, and AMS, with the overall response increasing as the gas concentration increased from 1 ppm to 10 ppm. Distinct differences were observed among the three sensor types in response amplitude, response and recovery dynamics, and concentration dependence, providing complementary temporal information for subsequent array-based recognition. To enable reliable signal acquisition, an STM32F103-based signal acquisition and processing circuit was further developed, incorporating constant-voltage excitation, current sensing, signal amplification, and signal conditioning. Based on the acquired dynamic response curves, a three-branch multi-scale one-dimensional residual network was employed for gas classification. Multi-scale convolution, residual connections, and squeeze-and-excitation (SE) channel attention were integrated to extract discriminative temporal features from the gas response process. Across three random seeds, the model achieved mean test-set accuracy, precision, recall, and F1-score values of 95.42% ± 0.34%, 95.46% ± 0.32%, 95.45% ± 0.36%, and 95.39% ± 0.35%, respectively. These results demonstrate that Au/Pd modification enhances the complementary and differential responses of the rGO/SnO_2_ sensor array toward sulfur-containing VOCs, while the integration of the sensor array with deep learning enables effective recognition of the three target gases. This work provides an experimental basis for the integrated implementation of gas sensing, signal acquisition, and intelligent recognition.

## 1. Introduction

With the accelerating trend of global population aging, the prevalence of age-related diseases such as osteoporosis continues to increase, posing a growing challenge to public health and quality of life. Osteoporosis is a typical age-related metabolic bone disease characterized by reduced bone mass, deterioration of bone microarchitecture, and increased bone fragility, which may lead to fragility fractures and impaired mobility in severe cases [1]. Osteoporosis is highly prevalent among older adults, particularly postmenopausal women. The decline in estrogen levels after menopause disrupts the dynamic balance between bone formation and bone resorption, making postmenopausal women a high-risk population for osteoporosis [2,3]. In addition, age-related declines in hormonal regulation, nutrient absorption, and physical activity further aggravate abnormalities in bone metabolism. Therefore, osteoporosis has become an important health concern among the elderly population. Currently, the clinical assessment of osteoporosis mainly relies on bone mineral density (BMD) measurements using dual-energy X-ray absorptiometry (DXA), together with an evaluation of fracture history and bone metabolic markers. However, DXA-based bone mineral density assessment has several limitations, including dependence on specialized equipment, relatively high cost, and limited suitability for early and dynamic monitoring [4]. Therefore, the development of a rapid, non-invasive, and low-cost auxiliary assessment technique is of great significance.

Volatile organic compounds (VOCs) are a class of carbon-containing organic molecules with high volatility. VOCs in human exhaled breath originate from multiple sources, among which a major proportion consists of volatile metabolites generated by endogenous metabolic processes. These metabolites are transported through the bloodstream to the lungs and subsequently exhaled through respiration. The major metabolic processes involved include lipid oxidation, protein degradation, and gut microbial metabolism [5]. Therefore, changes in the composition and concentrations of VOCs in exhaled breath can, to some extent, reflect an individual’s metabolic state and health status. Currently, exhaled VOC analysis has shown considerable potential for the auxiliary detection of diseases such as diabetes, lung cancer, and liver diseases [6,7,8]. Therefore, the analysis of exhaled VOCs associated with abnormal bone metabolism may provide useful information for exploring potential non-invasive assessment approaches for osteoporosis [9]. Recent clinical research has reported associations between sulfur-containing VOCs in exhaled breath and osteoporosis risk. In a study involving postmenopausal women, Sukul et al. [3] observed elevated concentrations of dimethyl sulfide (DMS), allyl methyl sulfide (AMS), and butanethiol in individuals at high risk of osteoporosis. The reported median breath concentrations of DMS, AMS, and butanethiol were 14.8, 4.30, and 1.21 ppbV in the discovery cohort, and 8.70, 2.30, and 0.50 ppbV in the independent validation cohort, respectively. These findings provide a scientific rationale for selecting these sulfur-containing VOCs as target analytes in the present study. However, their disease specificity and clinical diagnostic utility have not been fully established, and they should therefore be regarded as potential osteoporosis-associated breath markers rather than validated disease-specific biomarkers. Mass spectrometry provides high sensitivity and accuracy for detecting volatile sulfur compounds, although it often relies on specialized equipment and sample preparation. Gas sensor arrays, on the other hand, are relatively simple and have potential for portable applications. In this work, a Au/Pd-modified rGO-SnO_2_ sensor array was combined with pattern recognition to investigate the detection and discrimination of sulfur-containing VOCs. Zhou et al. [10] examined the relationship between VOC exposure and bone health and found that exposure to multiple VOCs was associated with lower bone mineral density. Although their study analyzed urinary VOC metabolites rather than exhaled breath, it provides further evidence of a possible link between VOC exposure and bone health.

Among various gas-sensing technologies, chemiresistive gas sensors offer advantages such as compact size, simple structure, ease of integration, and suitability for array construction, showing promising application potential for the detection of VOCs in exhaled breath. Chemiresistive gas sensors typically detect target gases through changes in electrical resistance caused by gas adsorption and desorption on the sensing material surface. Metal-oxide-based chemiresistive gas sensors possess abundant active sites, strong gas adsorption capability, and excellent chemical stability. However, metal oxides generally require relatively high operating temperatures and often exhibit relatively slow response and recovery kinetics. In contrast, graphene and its derivatives possess an extremely large specific surface area and ultrahigh carrier mobility, enabling highly sensitive gas detection at room temperature. However, the relatively high chemical inertness of the graphene surface results in an insufficient number of active adsorption sites, leading to weak gas adsorption capability and poor selectivity [11].

Besides conventional metal oxide semiconductors, various sensing materials and detection methods have been explored. Almaev et al. [12] investigated Er-doped ZnGa_2_O_4_ ceramics for methane detection and found that Er doping enhanced the sensing response. In addition to resistance-based sensing, Grigorjeva et al. [13] reported changes in the luminescence intensity of ZnO coatings under different oxygen concentrations, suggesting an alternative approach to gas detection. These studies demonstrate the different possibilities for gas sensing, while rGO/SnO_2_-based chemiresistive sensors provide a simple means of detecting gases through changes in electrical resistance.

Combining different sensing materials is an effective strategy for performance optimization, particularly through the construction of metal oxide–graphene composite sensing structures. Studies have shown that integrating the advantages of different materials in terms of electrical conductivity, gas adsorption capability, and surface reactivity can improve sensor sensitivity, response speed, stability, and low-temperature sensing performance [14]. In addition, functional modification of the sensing devices can further enhance selectivity and accelerate the response, thereby improving the adsorption capability toward target VOCs and providing a basis for constructing sensor arrays with differentiated response characteristics [15,16].

Reduced graphene oxide (rGO) is a two-dimensional carbon nanomaterial obtained by chemically, thermally, or electrochemically reducing graphene oxide. It possesses excellent electron transport capability, a large specific surface area, abundant defect sites, and good chemical stability [17]. However, its surface chemical activity and gas selectivity remain limited [18]. In contrast, tin oxide (SnO_2_), as a typical n-type metal oxide sensing material, possesses abundant surface adsorption sites, excellent gas reactivity, and good chemical stability, making it highly suitable for gas-sensing applications [19]. However, SnO_2_ also faces several challenges, including a relatively high operating temperature and poor selectivity, which may adversely affect the overall sensing performance of the sensor [20].

By constructing a composite structure of reduced graphene oxide (rGO) and metal oxide nanoparticles, the synergistic effects of the two materials can be fully exploited, thereby significantly enhancing gas adsorption and surface reaction capability while improving sensor sensitivity and response speed. The high electrical conductivity of rGO improves the charge transport properties of SnO_2_, whereas SnO_2_ provides enhanced gas adsorption capability [21]. The SnO_2_/rGO composite structure forms heterointerfaces that promote interfacial charge transfer, increase the number of effective active sites, and suppress material agglomeration, thereby achieving higher sensitivity, faster response, and improved low-temperature gas-sensing performance. These advantages also help reduce energy consumption and improve operational safety [22].

The aim of this study is to prepare SnO_2_ particles via a sol–gel method [23] and uniformly disperse reduced graphene oxide (rGO) through ultrasonic treatment to form an rGO ink. The SnO_2_ particles are subsequently combined with rGO to construct the basic gas-sensing devices. On this basis, Au and Pd noble-metal functionalization is employed to regulate the surface catalytic activity and interfacial charge-transfer characteristics of the sensing materials, thereby constructing a multichannel sensor array with differentiated gas-sensing responses for the detection of selected sulfur-containing VOCs, including dimethyl sulfide, allyl methyl sulfide, and butanethiol [3,24]. By combining unmodified, Au-modified, and Pd-modified rGO-SnO_2_ sensing devices, the array is designed to obtain complementary response characteristics toward these target gases. To address the problems of overlapping response curves among structurally similar VOCs and the limited selectivity of individual sensors, the multichannel sensor response data are further combined with a multibranch, multiscale ResNet model to extract discriminative features from the dynamic gas-response signals and establish a mapping relationship between sensor-array responses and gas categories, thereby enabling the intelligent classification and identification of similar VOCs. Furthermore, on the basis of the gas-sensing device study, a signal acquisition and processing circuit compatible with the sensors was designed to address the issues of low sensor output amplitude, susceptibility to noise and external environmental interference, and the limited suitability of conventional laboratory instruments for subsequent portable applications. Through functions including stable power supply, current variation detection, signal amplification, and filtering and conditioning, the circuit enables reliable acquisition of weak sensor response signals. Microcontrollers such as STM32 are further employed to accomplish data reading, processing, transmission, and display. In this study, differentiated sensor-array responses, dynamic signal recognition, and dedicated signal acquisition hardware are combined to investigate an integrated sensing approach for selected sulfur-containing VOCs. By integrating gas-sensing material composites and noble-metal functionalization, multichannel sensor array construction, ResNet-based intelligent recognition, and signal acquisition and processing hardware design, this study provides an experimental foundation and technical support for the detection and classification of sulfur-containing VOCs potentially associated with osteoporosis risk, as well as for the subsequent development of portable intelligent sensing systems.

## 2. Materials and Methods

### 2.1. Overall Sensor Structure

In this study, silicon wafers covered with a 300 nm SiO_2_ layer were used as the substrates for gas sensors. Reduced graphene oxide (rGO) and SnO_2_ sensing layers were sequentially constructed on the substrate surface. On this basis, Au and Pd noble metals were separately used for surface functionalization to regulate the surface activity and interfacial charge-transfer characteristics of the sensing materials, thereby constructing a multichannel sensor array with differentiated gas-sensing response characteristics. Three types of sensors, namely rGO/SnO_2_, Au-rGO/SnO_2_, and Pd-rGO/SnO_2_, were fabricated. Ten devices were prepared for each structure, giving a total of 30 devices, in order to reduce the influence of fabrication differences among individual devices on the experimental results and for subsequent repeatability and stability evaluation.

### 2.2. Preparation of the Reduced Graphene Oxide Sensing Layer

First, the Si/SiO_2_ substrates were cleaned sequentially with acetone, isopropanol, absolute ethanol, and deionized water to remove surface particles, organic contaminants, and residual impurities, followed by drying. Subsequently, a reduced graphene oxide (rGO) dispersion with a concentration of 1 mg mL^−1^ was prepared, and ultrasonic treatment was applied to promote the uniform dispersion of rGO nanosheets and reduce agglomeration. To improve the adhesion of rGO to the silicon wafer surface, the silicon wafers were first immersed in an (3-Aminopropyl)triethoxysilane (APTES) solution for surface functionalization. After treatment, the substrates were cleaned and dried, followed by further heating for 1 h. Subsequently, 6 μL of the rGO dispersion was pipetted and drop-cast onto the sensing area of the APTES-treated silicon wafer, allowing the rGO to be deposited in the central region of the wafer. After drying, an rGO sensing layer was formed. The detailed fabrication process of the rGO-modified Si/SiO_2_ substrate is illustrated in Figure S1 of the Supplementary Materials.

### 2.3. Preparation of the SnO_2_ Sensing Layer

**Figure 1:**
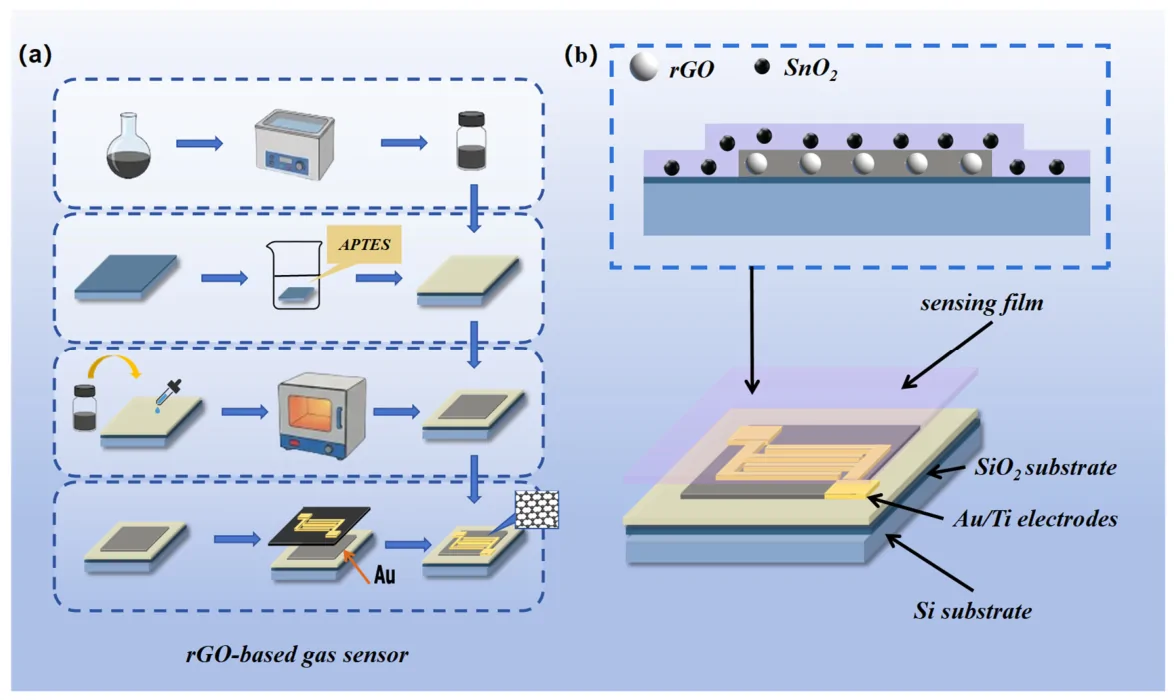
Schematic illustration of the rGO-SnO_2_ gas sensor: (**a**) fabrication process and (**b**) device structure.

As shown in Figure 1, a SnO_2_ thin film was further prepared on the surface of the rGO sensing layer. The tin oxide precursor solution was prepared using a sol–gel method. First, tin tetrachloride was dissolved in ethylene glycol (EG) at 80 °C under magnetic stirring. The solution was continuously stirred at 80 °C for 3 h to undergo the precursor reaction. Subsequently, the temperature was increased to 120 °C, and the solution was stirred for another 3 h to complete the hydrolysis reaction. Finally, glycerol was added to the solution under continuous stirring to adjust the viscosity and film-forming properties of the precursor solution, thereby obtaining the SnO_2_ precursor solution for subsequent spin coating. The prepared SnO_2_ precursor solution was then spin-coated onto the surface of the rGO sensing layer to form a uniform SnO_2_ thin film. After spin coating, the samples were first preheated at 100 °C for 10 min and subsequently annealed at 400 °C to further remove residual solvents and improve the stability of the SnO_2_ thin film.

### 2.4. Device Fabrication

The sensor electrodes were fabricated by thermal evaporation deposition. First, a corresponding metal shadow mask was prepared according to the predesigned electrode dimensions and structure, and the samples were fixed at the designated positions of the mask. After the evaporation chamber reached the required vacuum level, a 10 nm Ti adhesion layer and a 50 nm Au electrode layer were sequentially deposited to form Ti/Au interdigitated electrodes. The Ti layer was mainly used to improve the adhesion between the Au electrode and the substrate, while the Au layer served as the conductive electrode for stable acquisition of the sensor electrical signals. During the deposition process, the evaporation rate of the metal materials was controlled by adjusting the heating current of the evaporation source, with the deposition rate maintained at 0.5 Å/s, allowing the metal vapor to be transported under vacuum and deposited onto the sample surface through the shadow mask, thereby forming the designed interdigitated electrodes. To further regulate the gas-sensing characteristics of the sensors, a Au or Pd modification layer with a thickness of approximately 1 nm was deposited on the sensing regions of selected rGO/SnO_2_ devices. After deposition, the devices were placed on a hot plate for annealing, allowing the ultrathin noble-metal layer to undergo surface reconstruction during thermal treatment and thereby form nanoscale dispersed Au or Pd structures. Finally, three types of gas sensors with different sensing structures were fabricated, namely rGO/SnO_2_, Au-rGO/SnO_2_, and Pd-rGO/SnO_2_.

### 2.5. Gas-Sensing Performance Evaluation

The gas-sensing performance of the fabricated sensors was evaluated in a laboratory gas-testing chamber using a WS-30 gas sensor test system (Zhengzhou Winsen Electronics Technology Co., Ltd., Zhengzhou, China). For the dynamic response measurement of an individual device, the sensor was placed in a sealed test chamber and connected to a multimeter through probes. Under a constant bias condition, the current–time (I–t) response curves of the sensor at different gas concentrations were monitored in real time. In addition, a Winsen gas-testing platform equipped with an 18 L sealed chamber for gas detection was used. The platform enabled simultaneous measurement of the gas responses of a sensor array consisting of 30 sensors, allowing the response signals of different sensing units to be obtained under the same gas environment. The concentrations of the three VOCs were calculated using the following equation:

$$v_{x}=\frac{V_{c}\times C_{x}\times M_{x}}{24.45\times \rho_{x}\times p}\times 10^{-9}$$

where $v_{x}$ is the volume of the liquid VOC sample (mL), $C_{x}$ is the target gas concentration (ppm), $M_{x}$ is the molar mass of the liquid VOC (g/mol), $\rho_{x}$ is the density of the liquid VOC (g/cm^3^), $V_{c}$ is the volume of the sealed chamber (mL), and p denotes the purity of the liquid VOC. At 25 °C and 1 atm, the molar volume of an ideal gas is taken as 24.45 L mol^−1^ [25].

A specified volume of the target VOC liquid was injected into the heating region through the dedicated injection port of the chamber using a microsyringe. The target VOC was fully vaporized by heating, and the built-in fan in the chamber was used to accelerate gas mixing, allowing the target VOC to be distributed as uniformly as possible throughout the chamber. After the desired gas concentration was reached, the dynamic response signals of the sensor array were recorded. After each gas-sensing test, the chamber was opened and flushed with clean air to sufficiently remove the residual target gas while allowing the sensors to recover to their initial baseline states. During the measurements, gas-sensing parameters, including response amplitude, response time, and recovery time, were simultaneously recorded for each device.

### 2.6. Hardware Circuit Design

To achieve real-time and stable acquisition of gas sensor response signals and to provide continuous and reliable data for subsequent gas-response feature analysis and intelligent recognition, a signal acquisition and processing hardware system matched to the gas sensors was designed. The system mainly consists of a power supply module, sensor interface, current-sampling module, analog signal-conditioning module, STM32-based data acquisition and processing module, and data display and communication module. A constant-voltage bias current-detection mode was adopted, with a 5 V DC bias applied to the sensor test circuit and a sampling resistor connected in series with the sensor circuit. The signal amplified by the signal amplification circuit was acquired by the STM32. During each gas-sensing test cycle, the sensor was continuously biased and maintained at a stable baseline in clean air. The target VOC at the desired concentration was then introduced, and the dynamic response of the sensor signal was recorded. Subsequently, air was introduced to allow the sensor to recover to its initial state. This system provides a hardware and technical foundation for the subsequent development of portable detection systems.

### 2.7. Intelligent Recognition Algorithm

Dimethyl sulfide, allyl methyl sulfide, and butanethiol are all sulfur-containing volatile organic compounds (VOCs). Among them, dimethyl sulfide and allyl methyl sulfide both belong to the thioether class of sulfur-containing organic compounds and exhibit certain similarities in molecular structure and chemical properties. Therefore, the response characteristics of different sensors toward these gases may overlap to some extent. To further improve the discrimination of similar sulfur-containing VOCs, this study combines a multichannel sensor array with machine-learning algorithms for intelligent gas classification and recognition. The dynamic response curves obtained from the sensor array in dimethyl sulfide, allyl methyl sulfide, and butanethiol environments were used as the input data for the intelligent recognition model, and a corresponding gas classification dataset was established. Each complete dynamic response curve contains the continuous variation of the sensor from the initial baseline, through the gas exposure response, to the recovery stage, and can reflect temporal information such as response amplitude, response rate, curve shape, and recovery characteristics under different gases. During the experiments, each response curve was uniformly divided into a 30 s baseline stage, a 200 s target-gas response stage, and a 130 s recovery stage to ensure a consistent time scale across different experimental data.

Before model training, the raw response data were subjected to standardized preprocessing to reduce the influence of differences in experimental batches, device baselines, and signal amplitudes on the classification results. The response data were also normalized to ensure good comparability among signals with different amplitude ranges. Subsequently, the processed data were divided into training, validation, and test sets. The training set was used for model parameter optimization, the validation set was used for model structure selection and hyperparameter tuning, and the test set was used to evaluate the classification performance of the final model on unseen data. Compared with methods that use only a single maximum response value for classification, this study employed the complete dynamic response curves as model inputs, enabling the model to simultaneously capture multidimensional temporal information, including response amplitude, response rate, curve shape, and recovery rate, thereby providing a richer feature basis for distinguishing subtle differences among the three volatile organic compounds.

### 2.8. Material Characterization and Electrical Measurements

Scanning electron microscopy (SEM) was used to characterize the surface morphology of the sensing films in different devices, in order to observe the morphological characteristics of the rGO and SnO_2_ sensing layers on the substrate surface and to compare the changes in surface morphology before and after Au and Pd noble-metal modification. The structural characteristics of the materials were analyzed using Raman spectroscopy. The structural composition of the composite films was characterized based on the D and G bands of reduced graphene oxide and the characteristic vibrational peaks associated with SnO_2_. X-ray photoelectron spectroscopy (XPS) was employed to analyze the surface elemental composition and chemical states of the different sensing materials. The survey spectra were used to determine the presence of C, O, Sn, Au, and Pd in the samples, while the high-resolution C 1s, O 1s, Sn 3d, Au 4f, and Pd 3d spectra were analyzed to investigate the chemical bonding states and valence-state variations of the corresponding elements, thereby further verifying the rGO/SnO_2_ composite structure and the effectiveness of Au and Pd noble-metal modification. To further investigate the electrical performance of the sensor devices, electrical measurements were conducted on devices with different structures.

## 3. Results and Discussion

### 3.1. Material Structure and Morphology Analysis

**Figure 2:**
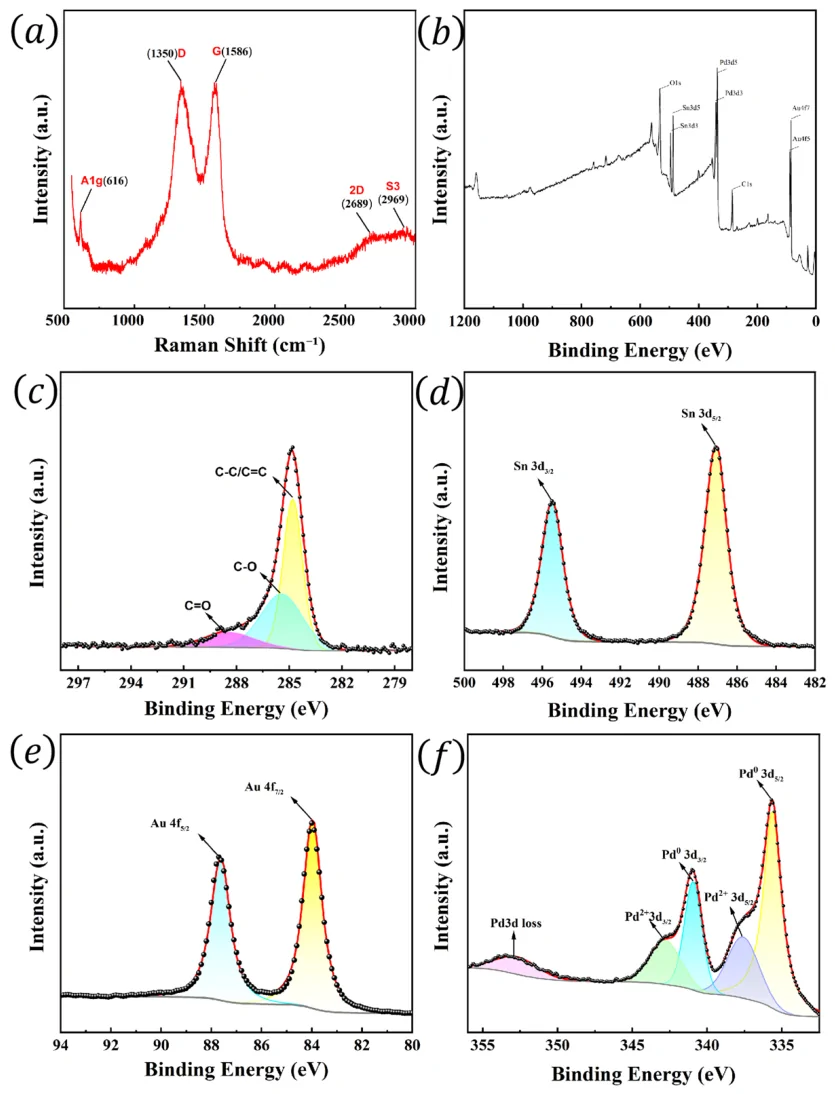
Raman and XPS characterization of the Au/Pd-modified rGO-SnO_2_ sensing devices: (**a**) Raman spectrum; (**b**) XPS survey spectrum; high-resolution XPS spectra of (**c**) C 1s, (**d**) Sn 3d, (**e**) Au 4f, and (**f**) Pd 3d. The black dots represent the raw experimental data, the red solid line is the overall fitted curve, and the colored filled regions are the deconvoluted sub-peaks.

The Raman spectrum of the sensing film is shown in Figure 2a. A distinct Raman band was observed at approximately 616 cm^−1^, which can be assigned to the A_1_g vibrational mode of rutile SnO_2_, confirming the presence of SnO_2_ in the sensing layer [26]. Two prominent Raman bands were observed at approximately 1350 and 1586 cm^−1^, corresponding to the D and G bands of reduced graphene oxide (rGO), respectively. The D band, typically located at approximately 1350 cm^−1^, is a defect-activated Raman band. In an ideal sp^2^ carbon lattice with high structural periodicity and integrity, the corresponding scattering process is restricted by the Raman selection rules. When defects, edges, or disordered regions are present in the graphene structure, they can provide the additional momentum required to activate phonons near the K point and enable their participation in the Raman scattering process [27]. The G band observed at approximately 1586 cm^−1^ originates from the in-plane E_2_g symmetric stretching vibration of sp^2^-hybridized carbon atoms and is associated with the first-order scattering of E_2_g phonons. The simultaneous presence of the D and G bands confirms the characteristic rGO structure in the sensing film.

The intensity ratio of the D and G bands I_D_/I_G_ was calculated to be approximately 1.04, indicating that the rGO retains a considerable degree of structural defects and disorder while preserving its sp^2^ carbon framework. These defects, edges, and disordered regions can provide potential active sites for gas adsorption and interfacial interactions, thereby facilitating charge transfer during the gas-sensing process [28]. In addition, a 2D band was observed at approximately 2689 cm^−1^. As a characteristic second-order Raman band of rGO, its spectral shape and relative intensity are associated with the layer structure and structural ordering of the carbon material. A D + G combination band was also observed at approximately 2969 cm^−1^, which can provide additional information regarding the defect degree and variations in the sp^2^ carbon structure.

As shown in Figure 2b–f, XPS characterization was used to analyze the elemental composition and chemical states on the sample surface. The survey spectrum of the sample contains C, Sn, Au, and Pd. The high-resolution C 1s spectrum can be deconvoluted into C=O, C-O, and C-C/C=C peaks, indicating the presence of oxygen-containing groups on the surface of the carbon substrate. The characteristic peaks of Sn 3d and Au 4f correspond to the characteristic orbital signals of Sn and metallic Au^0^, respectively. The fitting result of the Pd 3d spectrum shows that Pd exists in both metallic Pd^0^ and oxidized Pd^2+^ states. The detailed XPS peak parameters and elemental compositions of the Pd-modified, Au-modified, and pristine rGO-SnO_2_ sensors are summarized in Tables S1–S3 of the Supplementary Materials, respectively.

**Figure 3:**
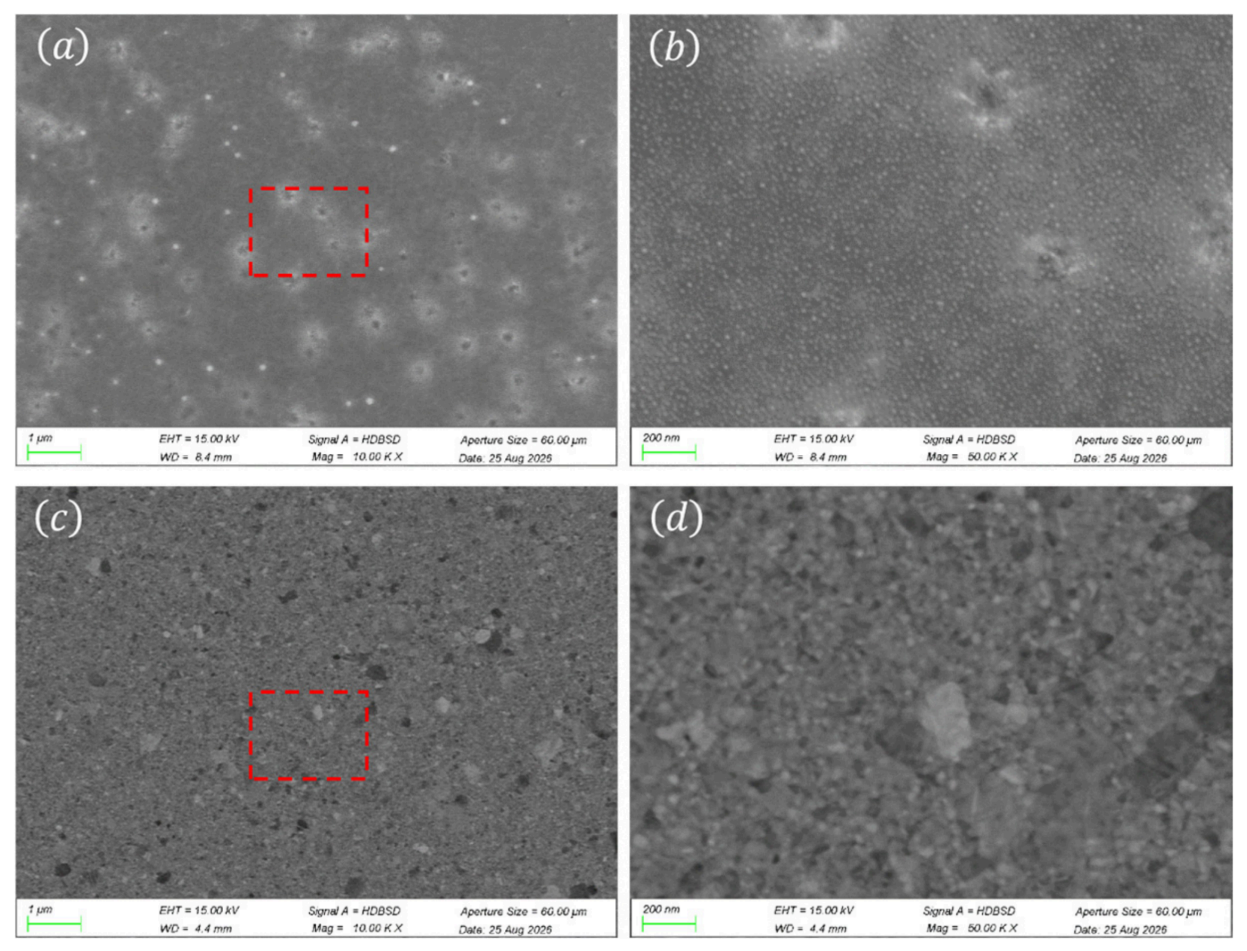
SEM micrographs of (**a**,**b**) rGo-SnO_2_-Au, (**c**,**d**) rGo-SnO_2_-Pd sensors. The red dashed squares in panels (**a**) and (**c**) indicate the selected areas for high-magnification observation shown in panels (**b**) and (**d**), respectively.

The surface morphology of the sensing devices was characterized and analyzed using scanning electron microscopy (SEM). Figure 3a–d presents SEM images of the Au- and Pd-modified rGO/SnO_2_ devices at different magnifications. It can be observed that both device surfaces exhibit distinct nanoparticle features. The low-magnification SEM images indicate that both the Au and Pd modification layers can effectively cover the surface of the sensing material. At higher magnifications, relatively dense and uniformly distributed nanoparticles can be observed on the surface of the Au-modified sample, whereas the Pd-modified sample exhibits more pronounced particulate features and localized agglomeration. These results indicate that, after thermal evaporation and subsequent annealing, both Au and Pd can form nanoscale metal particles on the rGO/SnO_2_ surface, although certain differences exist in their surface morphology and particle aggregation states. The SEM images of the pristine and noble-metal-modified rGO-SnO_2_ sensing films are presented in Figure S2, while the corresponding EDS elemental mapping results of the Au- and Pd-modified sensors are provided in Figures S3 and S4 of the Supplementary Materials, respectively.

### 3.2. Gas-Sensing Performance of Au/pd-Modified rGO/SnO_2_ Gas Sensors

The gas-sensing performances of three devices, namely rGO-SnO_2_, rGO-SnO_2_-Au, and rGO-SnO_2_-Pd composite thin-film devices, were compared. The three devices exhibited different gas-sensing characteristics toward 1-butanethiol, dimethyl sulfide, and allyl methyl sulfide. Their response and recovery times were further investigated, as these parameters are important for evaluating the dynamic performance and reliability of gas sensors. The response time of a gas sensor refers to the time required for its output signal to change relative to a reference condition, such as clean air, and reach a stable response state after exposure to the target gas. Three VOCs at a constant concentration of 10 ppm were used as analytes in the experiments.

**Figure 4:**
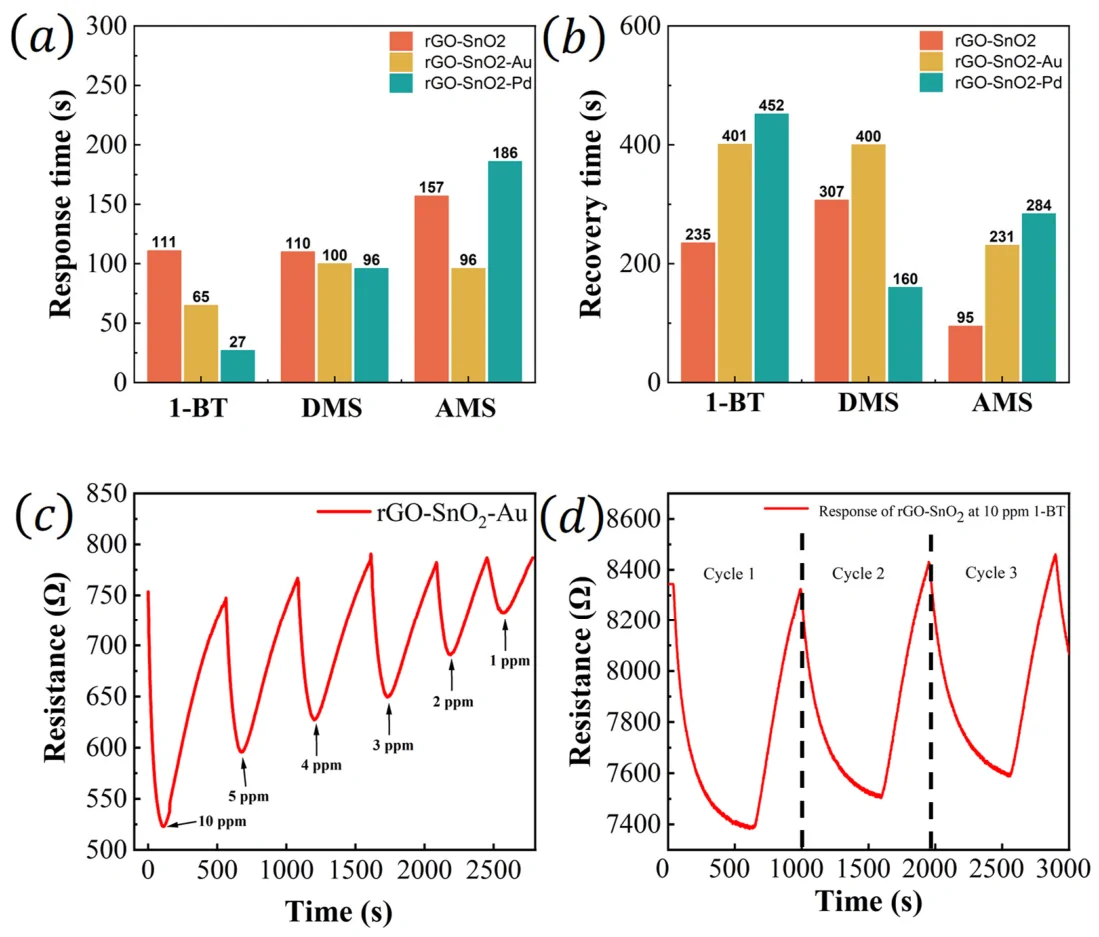
Gas-sensing performance of the rGO-SnO_2_-based sensor array. (**a**) Response times of the rGO-SnO_2_, rGO-SnO_2_-Au, and rGO-SnO_2_-Pd sensors toward 1-butanethiol (1-BT), dimethyl sulfide (DMS), and allyl methyl sulfide (AMS); (**b**) recovery times of the three sensors toward 1-BT, DMS, and AMS; (**c**) dynamic responses of the rGO-SnO_2_-Au sensor to different concentrations of 1-BT; and (**d**) repeatability of the rGO-SnO_2_ sensor toward 10 ppm 1-BT over three consecutive sensing cycles.

As shown in Figure 4a,b. The experimental results showed that, for 1-butanethiol, the time required for the rGO-SnO_2_-Au and rGO-SnO_2_-Pd devices to reach the minimum resistance was generally shorter than that of the rGO-SnO_2_ device without noble-metal modification, indicating that noble-metal modification facilitated the response of the devices to 1-butanethiol. Among them, the rGO-SnO_2_-Pd device exhibited a relatively fast response but a longer recovery time. For dimethyl sulfide, all three devices produced relatively pronounced responses within a short period, whereas their recovery times differed significantly. The rGO-SnO_2_-Au device exhibited the longest recovery time, while the rGO-SnO_2_-Pd device recovered more rapidly, showing a better dynamic balance between response and recovery. For allyl methyl sulfide, the response times of all three devices were longer than those observed for 1-butanethiol and dimethyl sulfide. The two noble-metal-modified devices required relatively long periods to complete the recovery process, particularly the Pd-modified device, whereas the unmodified device exhibited a faster recovery. The detailed response and recovery characteristics of the three sensor types toward BT, DMS, and AMS are provided in Figure S5.

Therefore, noble-metal modification of the device surface not only altered the gas adsorption and reaction rates but also significantly affected the gas desorption process. The three devices exhibited distinct response and recovery times toward the three VOCs. The Pd-modified device promoted the response process toward dimethyl sulfide and 1-butanethiol, whereas the Au-modified device exhibited relatively long recovery times for all three gases. These distinct dynamic temporal characteristics among the three devices also provided discriminative data for subsequent gas-species recognition.

As shown in Figure 4c, the rGO-SnO_2_-Au sensor exhibited distinct resistance changes upon exposure to 1-BT at different concentrations. The sensor also showed repeated response–recovery behavior throughout the measurements. Figure 4d presents the repeatability test of the sensor upon exposure to 10 ppm 1-butanethiol. Three consecutive tests were conducted under the same experimental conditions, and the sensor exhibited good repeatability and stability over the three response–recovery cycles.

**Figure 5:**
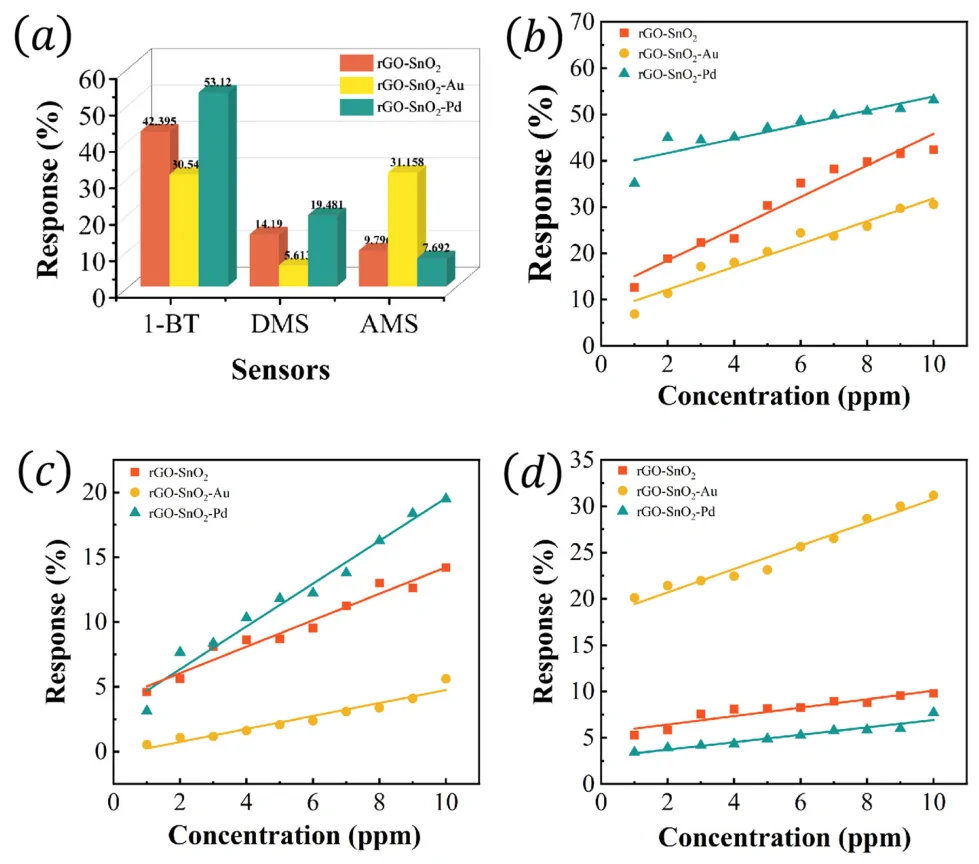
Response characteristics of the rGO-SnO_2_-based sensor array. (**a**) Comparison of the responses of the rGO-SnO_2_, rGO-SnO_2_-Au, and rGO-SnO_2_-Pd sensors toward 10 ppm 1-BT, DMS, and AMS; calibration curves of the three sensor types toward (**b**) 1-BT, (**c**) DMS, and (**d**) AMS.

To compare the gas-sensing responses and quantitative detection capabilities of the three devices toward different sulfur-containing VOCs, gas-sensing tests were conducted on the rGO-SnO_2_, rGO-SnO_2_-Au, and rGO-SnO_2_-Pd devices at different gas concentrations. As shown in Figure 5a, at a concentration of 10 ppm, the three devices exhibited distinctly different response characteristics toward 1-butanethiol (1-BT), dimethyl sulfide (DMS), and allyl methyl sulfide (AMS). Among them, the rGO-SnO_2_ and rGO-SnO_2_-Pd devices exhibited the highest responses to 1-BT, with response values of 42.395% and 53.12%, respectively, whereas the rGO-SnO_2_-Au device showed a relatively high response to AMS, with a response value of 31.158%. The differentiated response characteristics of the three devices toward different target gases indicate that noble-metal modification can alter the gas-sensing responses of the devices toward different sulfur-containing VOCs, providing a basis for distinguishing different gases using a sensor array.

To further evaluate the quantitative detection capability of the three devices, their responses toward the three VOCs were measured over a concentration range of 1–10 ppm. The response values at each concentration were extracted, and calibration curves were established using gas concentration as the horizontal axis and sensor response as the vertical axis. Figure 5b–d show the concentration–response relationships of the three devices toward 1-BT, DMS, and AMS, respectively. As the target gas concentration increased, the response values of the three devices generally increased, indicating an evident concentration dependence of their responses. Within the tested concentration range, linear fitting was performed for each dataset. Following the order of rGO-SnO_2_, rGO-SnO_2_-Au, and rGO-SnO_2_-Pd in Figure 5b–d, the coefficients of determination, R^2^, were 0.95807, 0.95104, and 0.82344; 0.96335, 0.93954, and 0.97214; and 0.88300, 0.96868, and 0.92530, respectively. The corresponding calibration curves for the three sensors toward BT, DMS, and AMS are provided in Figure S6. Additionally, the effects of relative humidity on the sensing responses and the stability of the rGO-SnO_2_ sensor after four months of storage are presented in Figures S7 and S8, respectively. A comparison of the proposed sensors with commercially available and previously reported gas sensors is provided in Table S10 of the Supplementary Materials.

$$\mathrm{Response}=\frac{\left(R_{0}-R_{g}\right)}{R_{0}}\times 100\%$$

where $R_{0}$ is the baseline resistance before exposure to the target gas, and $R_{g}$ is the resistance of the sensor after reaching a stable state upon exposure to the target gas.

$$\mathrm{Sensitivity}=k=\frac{\Delta Response}{\Delta C}$$

Among them, the rGO-SnO_2_-Pd device exhibited the highest linear fitting degree for the concentration–response relationship toward DMS, with *R*^2^ = 0.97214 while the rGO-SnO_2_-Au device also showed a good linear fitting result toward AMS, with *R*^2^ = 0.96868. These results indicate that most sensor–gas combinations exhibited good concentration–response linearity within the range of 1–10 ppm and could stably reflect variations in the target gas concentration. In contrast, the fitting coefficients of determination for rGO-SnO_2_-Pd toward 1-BT and rGO-SnO_2_ toward AMS were relatively lower, at 0.82344 and 0.88300, respectively, with the experimental data points showing certain deviations from the linear fitting curves. A schematic representation of the dynamic gas-sensing measurement system is provided in Figure S9.

### 3.3. Gas-Sensing Mechanism

**Figure 6:**
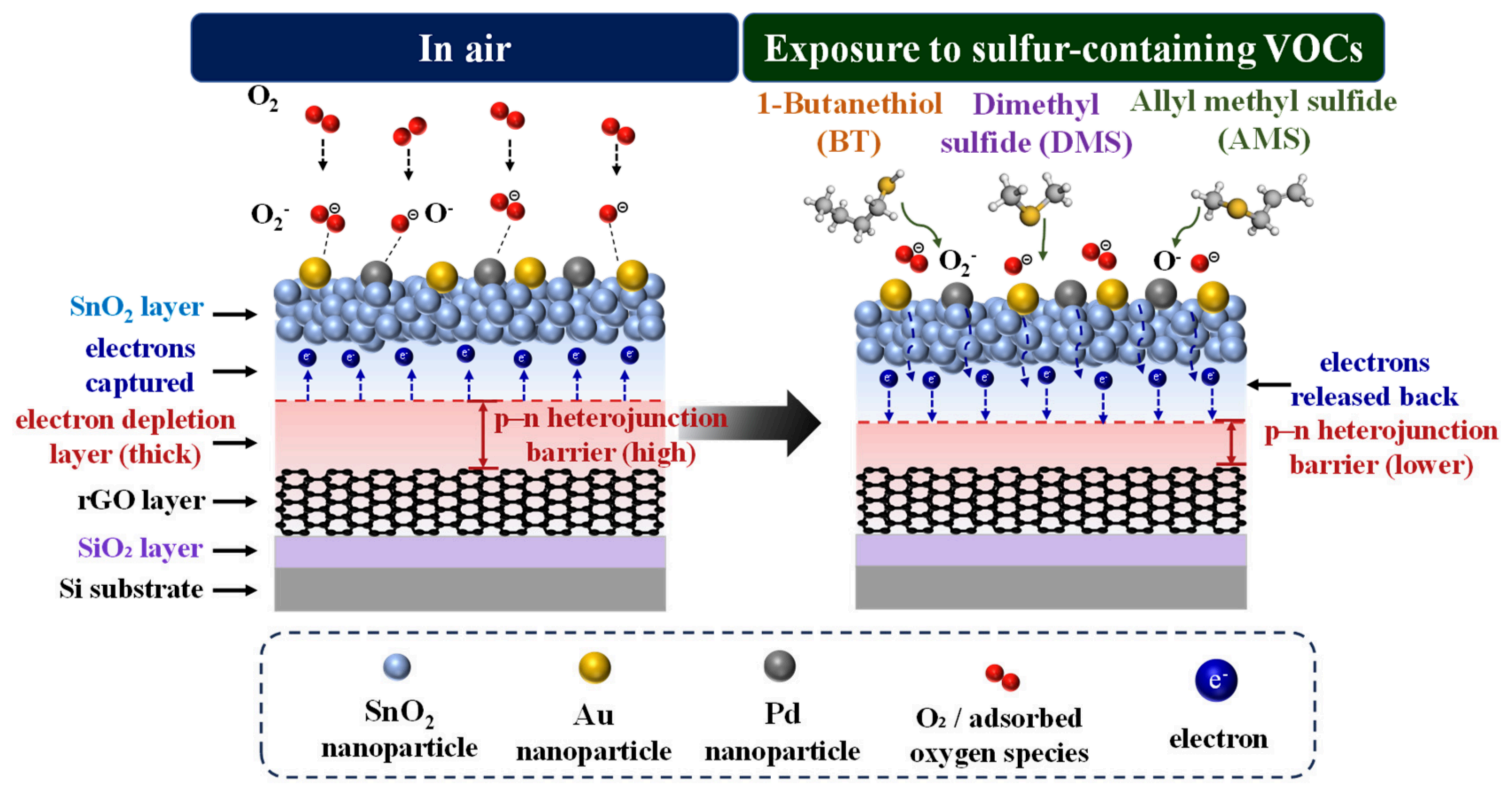
Schematic illustration of the gas-sensing mechanism of the Au/Pd-modified rGO-SnO_2_ sensor.

As shown in Figure 6, the gas-sensing devices constructed in this study consist of a silicon substrate, an rGO layer, a SnO_2_ sensing layer, and noble-metal surface modification layers such as Au and Pd. The silicon substrate serves as the structural support and insulating substrate of the device, whereas the gas-sensing process mainly occurs within the sensing layer. Since all three types of devices are based on the rGO/SnO_2_ structure, they share a similar fundamental gas-sensing mechanism. SnO_2_ is a typical n-type wide-bandgap semiconductor. When reducing sulfur-containing VOCs, such as dimethyl sulfide, allyl methyl sulfide, and butanethiol, come into contact with SnO_2_, the gas molecules undergo physical and chemical adsorption and subsequently participate in electron-exchange processes at surface defect-related active sites. During this process, the reducing VOC molecules react with the adsorbed oxygen species originally formed by oxygen adsorption from air, causing the electrons previously trapped by the adsorbed oxygen species to be released back into SnO_2_. As a result, the electron concentration increases, the surface depletion layer becomes narrower, and the electrical resistance of the device decreases [29,30,31].

When a SnO_2_ sensing layer is introduced onto reduced graphene oxide, the gas response is determined not only by the surface reactions occurring on SnO_2_ but also by interfacial charge transfer between rGO and SnO_2_. Reduced graphene oxide possesses a two-dimensional sheet-like structure, high carrier transport capability, and abundant defect sites, including edges, vacancies, and residual oxygen-containing functional groups, which provide additional sites for gas adsorption and charge exchange [32]. Under ambient conditions, rGO exhibits p-type semiconducting behavior, whereas SnO_2_ is an n-type semiconductor. When the two materials come into contact, a p–n heterojunction is formed. Previous studies have shown that the heterostructure formed between rGO and SnO_2_ can promote interfacial charge transfer and thereby influence the gas-sensing response [22]. Upon exposure to the target VOCs, not only is the intrinsic conductivity of SnO_2_ altered, but the interfacial potential barrier at the rGO/SnO_2_ heterojunction is also modulated, allowing relatively small surface chemical changes to be converted into more pronounced resistance variations. The overall resistance of the device decreases after exposure to the target gases, indicating that the increase in conductivity associated with the SnO_2_-related n-type conduction channel and the reduction in the heterojunction interfacial barrier play dominant roles. The rGO increases the number of available surface adsorption sites, shortens the interfacial carrier transport pathways, and further amplifies gas-induced charge variations through the heterojunction. Therefore, the rGO/SnO_2_ composite used in this study combines the favorable charge-transfer capability of two-dimensional carbon materials with the strong surface chemical activity of metal oxides.

The surfaces of Au and Pd nanoparticles can provide adsorption and active sites for oxygen molecules and VOC molecules, such as dimethyl sulfide, allyl methyl sulfide, and butanethiol, that are different from those on SnO_2_, thereby accelerating the redox reactions occurring on the SnO_2_ surface. In addition, charge redistribution at the Au/Pd-SnO_2_ interface induces an electronic sensitization effect and modulates the surface depletion layer, thereby enhancing the gas-induced resistance changes. The activated adsorbed species can migrate from the noble-metal sites to the adjacent SnO_2_ surface through a spillover-assisted pathway, thereby improving the sensitivity of the sensor toward VOCs such as butanethiol, allyl methyl sulfide, and dimethyl sulfide [33,34].

### 3.4. Hardware Circuit of the Gas-Sensing Test Platform

**Figure 7:**
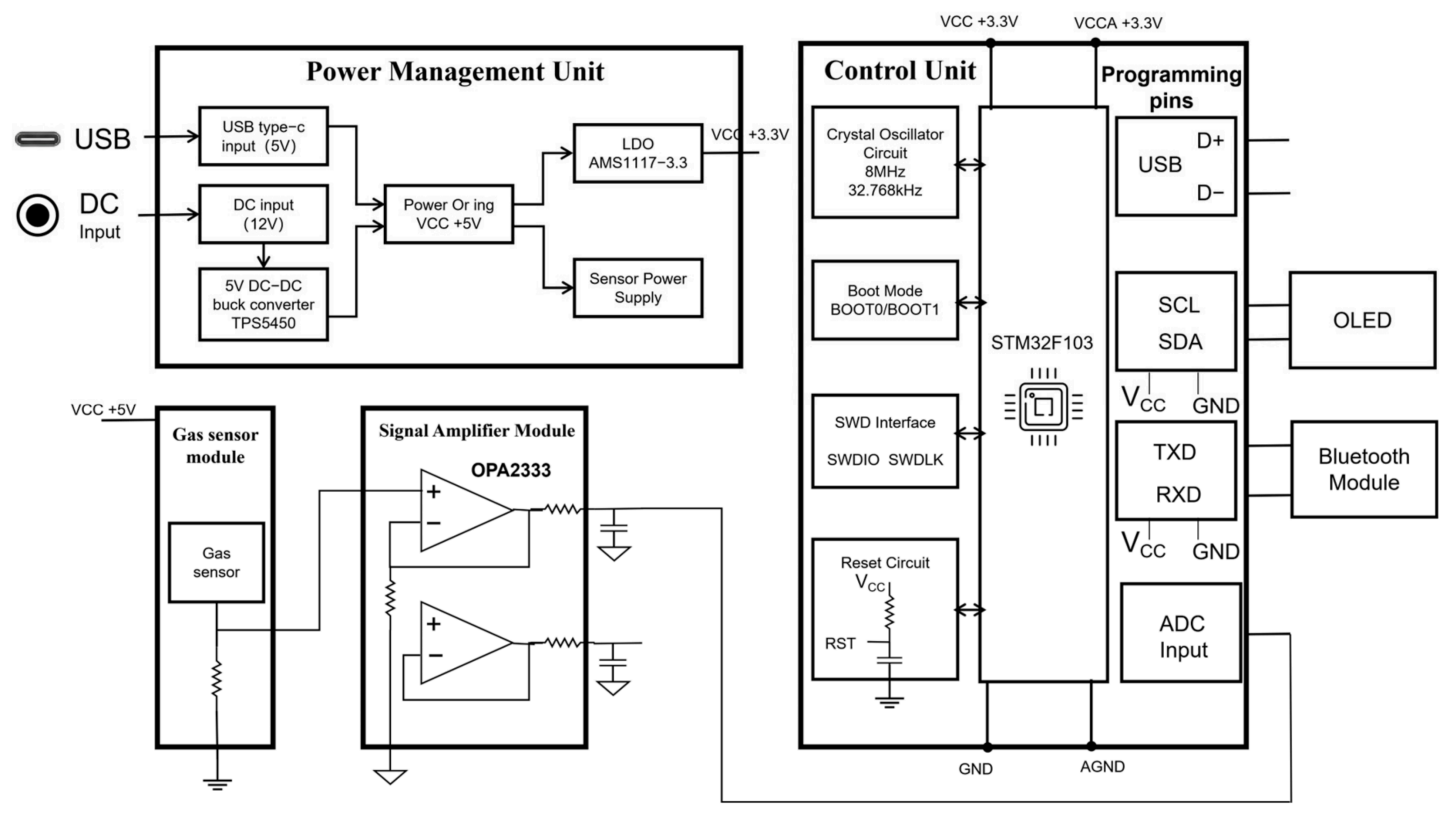
Block diagram of the hardware circuit system for the gas sensor.

In this study, considering the characteristics of kΩ-level resistance and μA-level current variations of rGO/SnO_2_ and Au/Pd-modified gas-sensing devices, a signal detection system based on constant-voltage excitation and low-resistance current sampling was designed using JLCEDA. The detailed block diagram of the proposed system is shown in Figure 7. The system consists of a power management unit, a control unit, and a sensor signal reading unit. The control unit is responsible for acquiring the voltage signals of the sensors after signal conditioning, completing data processing and control, and outputting the detection results to the OLED and the serial port for communication transmission. The power management unit is responsible for providing a stable supply voltage for the entire system. A stable 5 V voltage was used to provide the operating bias voltage for the gas sensors, and a 100 Ω precision sampling resistor was used to convert the sensor current variations into mV-level voltage signals, thereby reducing the influence of the sampling circuit on the operating state of the sensors and realizing the detection of electrical signal variations on this basis.

The purpose of the sensor signal reading unit in this study is to digitize the resistance of the target gas sensor. To achieve the conversion between resistance and voltage, a 100 Ω sampling resistor was placed between ground and one electrode of the sensor, while the other electrode of the sensor was connected to the constant 5 V supply (VCC + 5V) from the power management unit, as shown in Figure 7. Because the resistance of the sampling resistor is relatively small, the voltage across its two terminals is also small, and the resistance of the sensor is much larger than that of the sampling resistor. For the 12-bit ADC of the STM32F103 microcontroller with a 3.3 V reference voltage, each digital count corresponds to approximately 0.8 mV. Therefore, the voltage signal across the sampling resistor corresponds to only several tens of ADC counts, and the curve corresponding to the small variations caused by the gas response is relatively coarse. To address this issue, a signal amplification circuit based on the OPA2333 was employed to condition the sensor signal, thereby improving the utilization of the ADC input range, signal resolution, and anti-interference capability, and providing more stable raw data for subsequent gas-response processing. For the power management unit, the entire circuit board was designed to be powered by USB, a 12 V DC supply, or a button cell. A USB Type-C interface is normally used to power the circuit board, and the acquired data are transmitted to a personal computer through USB 2.0. To provide a stable 5 V supply voltage, a power-supply decoupling and filtering circuit was added between the 5 V power supply and the sensor electrode. The complete circuit schematic and a photograph of the fabricated hardware circuit are provided in Figures S10 and S11, respectively.

### 3.5. Intelligent Gas Recognition Algorithm

**Figure 8:**
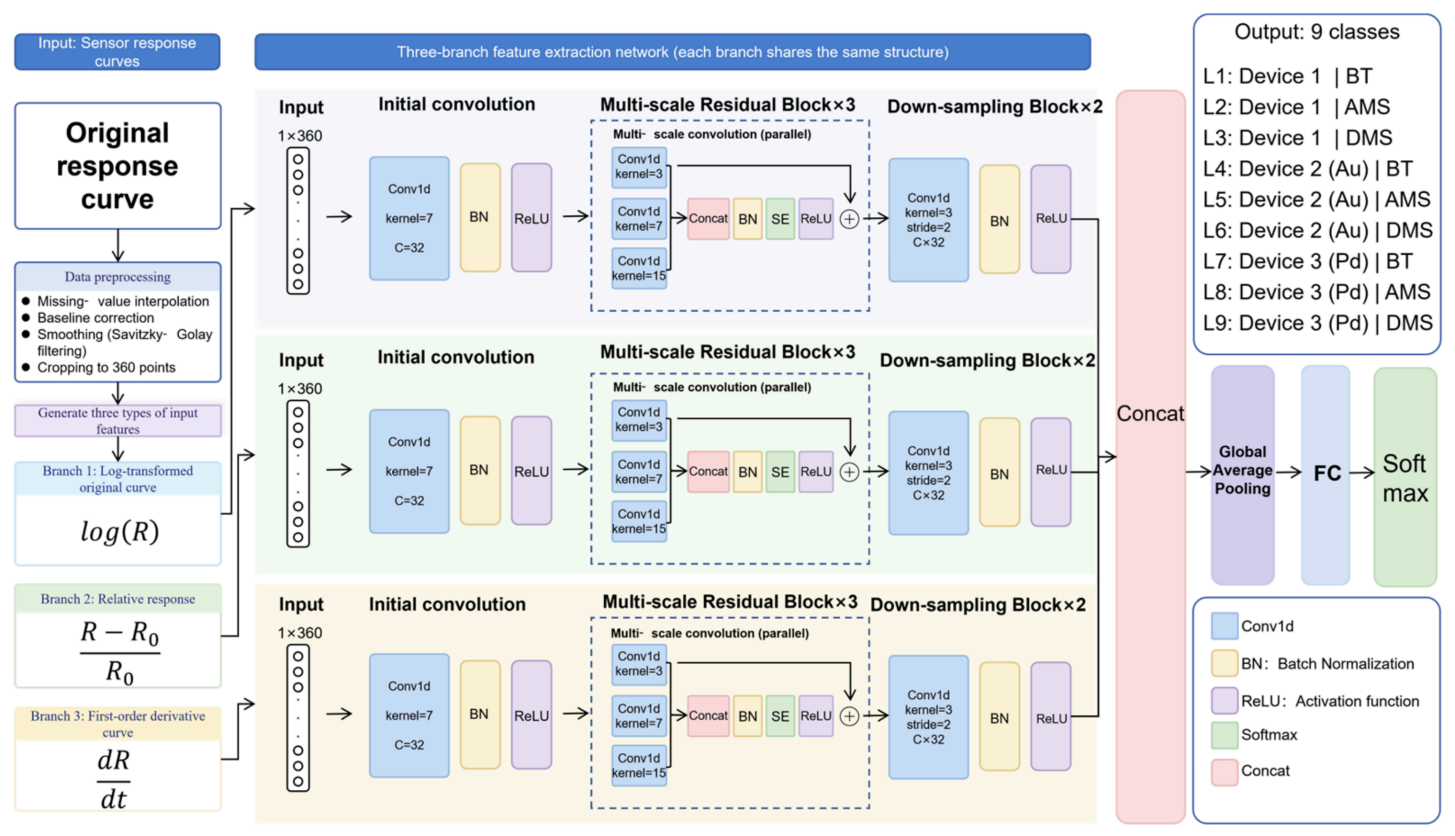
Architecture of the proposed three-branch multi-scale 1D-ResNet for gas classification.

To extract distinctive deep features from the dynamic response signals of gas sensors, a gas intelligent recognition model based on a residual neural network (Residual Neural Network, ResNet) was established in this study. As shown in Figure 8. The model takes the preprocessed sensor response sequences as input and extracts local variation features from the response curves through convolutional layers. The detailed data preprocessing workflow is illustrated in Figure S12 of the Supplementary Materials. Meanwhile, multiple residual blocks are established to progressively extract discriminative features from the response signals of different gases. The core structure of ResNet is the residual connection, in which the input features are fused with the features extracted by the convolutional layers through shortcut connections. This structure can effectively alleviate the vanishing-gradient and network-degradation problems caused by increasing network depth, enabling the model to maintain stable training in a deeper network architecture and extract multidimensional temporal features from the gas response curves [35]. The model automatically learns the comprehensive differences among different gases in terms of response amplitude, response process, dynamic variation trends, and recovery characteristics from the complete dynamic response curves, thereby achieving intelligent classification of the target gases.

**Figure 9:**
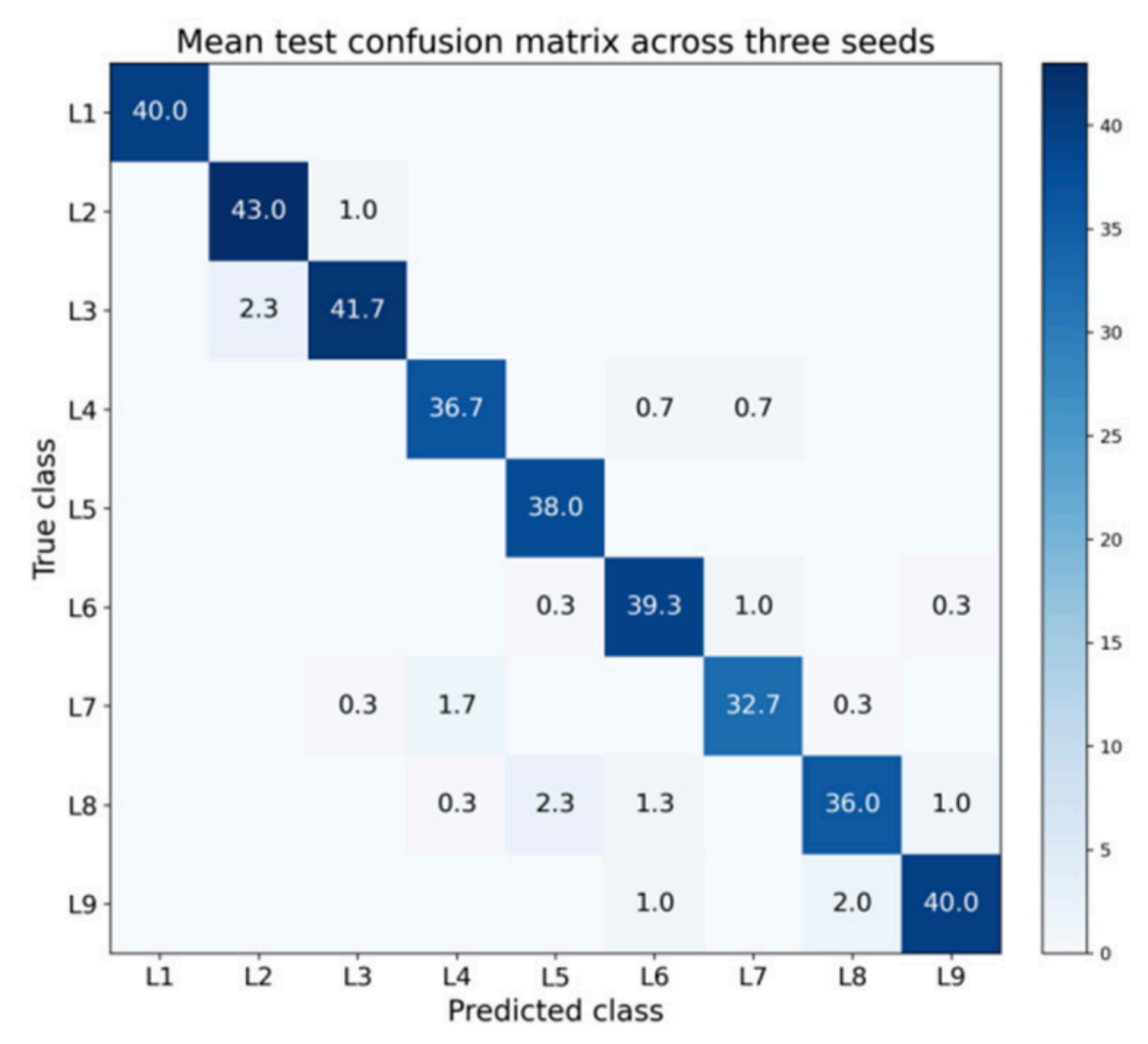
Mean confusion matrix of the three-branch multi-scale 1D-ResNet.

To further analyze the specific recognition performance of the model for the three gases, a confusion matrix was constructed based on the prediction results of the test set, as shown in Figure 9. The main diagonal of the confusion matrix represents the proportion of samples that were correctly classified, whereas the off-diagonal elements reflect the misclassification among different gases. Overall, most of the test samples are concentrated along the main diagonal of the confusion matrix, indicating that ResNet can effectively distinguish dimethyl sulfide, allyl methyl sulfide, and butanethiol. Among the three gases, butanethiol exhibits the highest classification accuracy, indicating that its response characteristics in the sensor array are more distinct from those of the other two gases. In contrast, a certain degree of misclassification occurs between dimethyl sulfide and allyl methyl sulfide. This may be related to the similarity in the response-curve shapes and dynamic variation trends of the two gases on some sensor devices, making it difficult to completely distinguish them using a single response amplitude. Nevertheless, ResNet can further extract high-dimensional features from subtle dynamic differences during the response process, thereby achieving a relatively high overall classification accuracy. The results of the confusion matrix indicate that combining the multidimensional response signals generated by the gas sensor array with deep-learning algorithms can effectively compensate for the limited selectivity of individual sensors, thereby improving the system’s discrimination capability for structurally similar sulfur-containing volatile organic compounds.

To further evaluate the classification performance of the proposed model, additional experiments were conducted under random curve-level splitting and exposure-run-based splitting. The classification results under these two data partitioning strategies are summarized in Table S4. Furthermore, the proposed model was compared with PCA-LDA, random forest, RBF-SVM, and 1D-CNN, and ablation experiments were performed to examine the contributions of different input channels and network components. The corresponding baseline comparison and ablation results are presented in Tables S5–S8 of the Supplementary Materials.

To quantitatively evaluate the recognition performance of the ResNet model for different target gases, Accuracy, Precision, Recall, and F1-score were used as the evaluation metrics for model classification performance in this study. To ensure the reliability of the experiments, three random-seed experiments were conducted using seeds of 13, 7, and 4. The test results are shown in Table 1. The ResNet model achieved a classification accuracy of 95.88% on the test set, while the Precision, Recall, and F1-score reached 95.87%, 95.92%, and 95.86%, respectively. The high classification performance indicates that although the three volatile organic compounds exhibit relatively similar response trends on some gas sensors, the complete dynamic response process still contains various distinctive features that can be used for gas classification. Through multilayer convolution and residual feature extraction, ResNet can comprehensively analyze features such as response amplitude, response rate, curve variation patterns, and recovery curves, thereby achieving effective recognition of different gases. Compared with methods based solely on steady-state response values or individual manually selected features, the ResNet-based recognition method reduces the dependence on manual feature selection and can automatically learn discriminative high-dimensional features directly from the raw response sequences.

The results demonstrate that combining differentiated gas-sensor responses with deep-learning algorithms can effectively improve the system’s discrimination capability for structurally similar volatile organic compounds, providing a feasible approach for constructing an integrated detection system that incorporates gas detection, signal acquisition, data processing, and intelligent recognition.

## 4. Conclusions

In summary, the combination of rGO and SnO_2_ produced a synergistic effect, and three types of gas sensors were constructed by further introducing Au and Pd noble-metal modifications. The sensing performances of these sensors toward butanethiol, dimethyl sulfide (DMS), and allyl methyl sulfide (AMS) were systematically evaluated. The results showed that all three types of devices exhibited measurable and distinguishable responses to the target gases. As the gas concentration increased from 1 to 10 ppm, the sensor responses generally increased, demonstrating good concentration-dependent characteristics. Among them, the pristine rGO/SnO_2_ device exhibited a relatively pronounced concentration-dependent response toward butanethiol, the Pd-modified device showed a good linear correlation with DMS concentration, and the Au-modified device also exhibited a good linear response to AMS. Noble-metal modification may modulate the interfacial reactions and charge-transfer processes between the rGO/SnO_2_ composite sensing material and different gas molecules, thereby generating differentiated gas-sensing response characteristics. In addition, noticeable differences in response and recovery times were observed among the different devices for the three target gases, providing additional dynamic feature information for multi-gas recognition. The sensor array composed of the three sensing materials was able to obtain complementary multidimensional response features and, when combined with an intelligent recognition algorithm, enabled effective discrimination of the three sulfur-containing VOCs. A comparison of the proposed sensing system with previously reported studies in terms of sensing materials, target gases, recognition methods, and hardware integration is provided in Table S9 of the Supplementary Materials. The proposed sensor array provides a potential platform for the detection of sulfur-containing VOCs in breath analysis. However, its applicability to osteoporosis screening or clinical diagnosis requires further validation using real breath samples and assessment of the diagnostic relevance of these VOCs.

## Figures and Tables

**Table 1:** Classification performance of the three-branch multi-scale 1D-ResNet.

Seed	Accuracy	Precision	Recall	F1-Score
13	95.05	95.11	95.05	95.01
7	95.88	95.87	95.92	95.86
4	95.33	95.40	95.37	95.31
Mean	95.42 (±0.34)	95.46 (±0.32)	95.45 (±0.36)	95.39 (±0.35)

## Data Availability

The data presented in this study are available on request from the corresponding author.

## Supplementary Materials

The following supporting information can be downloaded at: https://www.mdpi.com/article/10.3390/ma19214606/s1. Table S1: XPS peak parameters and elemental composition of the rGO-SnO_2_-Pd sensor; Table S2: XPS peak parameters and elemental composition of the rGO-SnO_2_-Au sensor; Table S3: XPS peak parameters and elemental composition of the rGO-SnO_2_ sensor; Table S4: Classification performance of the proposed model under different data partitioning strategies; Table S5: Classification performance of the proposed model and baseline methods under random curve-level splitting; Table S6: Classification results of the proposed model and baseline methods under exposure-run-based splitting; Table S7: Input-channel and network-module ablation results under random curve-level splitting; Table S8: Input-channel and network-module ablation results under exposure-run-based splitting; Table S9: Comparison of the proposed gas sensing system with previously reported studies; Table S10: Comparison of the proposed sensors with commercially available and previously reported gas sensors; Figure S1: Photographs illustrating the fabrication process of the rGO-based gas sensor, including sensing suspension preparation, suspension dispensing, APTES treatment, rGO dispersion preparation, rGO drop-casting, substrate modification, device fabrication, and SEM characterization; Figure S2: SEM images of the pristine and noble-metal-modified rGO-SnO_2_ sensing films at different magnifications: (a,b) pristine rGO-SnO_2_, (c,d) Au-modified rGO-SnO_2_, and (e,f) Pd-modified rGO-SnO_2_; Figure S3: EDS elemental mapping images of the rGO-SnO_2_-Au sensor: (a) elemental overlay and (b–f) individual elemental distribution maps of Si, O, Au, Sn and C, respectively; Figure S4: EDS elemental mapping images of the rGO-SnO_2_-Pd sensor: (a) elemental overlay and (b–h) individual elemental distribution maps of Si, Au, O, Sn, Pd, Ti, and C, respectively; Figure S5: Response and recovery characteristics of the rGO-SnO_2_-based sensor array toward sulfur-containing VOCs: (a–c) 1-BT, (d–f) DMS, and (g–i) AMS. The three columns correspond to rGO-SnO_2_, rGO-SnO_2_-Au, and rGO-SnO_2_-Pd sensors, respectively; Figure S6: Calibration curves of the responses of rGO-SnO_2_, rGO-SnO_2_-Au, and rGO-SnO_2_-Pd sensors toward different concentrations of butanethiol (first row), dimethyl sulfide (second row), and allyl methyl sulfide (third row); Figure S7: Effect of relative humidity on the sensing responses of pristine rGO-SnO_2_, rGO-SnO_2_-Au, and rGO-SnO_2_-Pd sensors toward 10 ppm 1-butanethiol (1-BT); Figure S8: Repeated response–recovery cycles of the rGO-SnO_2_ sensor after four months of storage under exposure to 10 ppm 1-BT; Figure S9: Dynamic Gas Sensing Test System; Figure S10: Schematic diagram of the hardware circuit; Figure S11: Photograph of the Fabricated Hardware Circuit; Figure S12: Workflow of sensor response preprocessing and three-channel feature construction; Algorithm S1: Three-branch multi-scale 1D-ResNet for sulfur-containing VOC classification; Algorithm S2: Multi-scale residual block; References [16,35,36,37,38,39,40,41,42] are cited in the Supplementary Materials.

## Abbreviations

VOCs	Volatile organic compounds
rGO	Reduced graphene oxide
SEM	Scanning electron microscopy
XPS	X-ray photoelectron spectroscopy
BMD	Bone mineral density
DXA	Dual-energy X-ray absorptiometry
ResNet	Residual neural network
ADC	Analog-to-digital converter
MCU	Microcontroller unit
