# Supplementary material for: Intelligent Recognition of Sulfur-Containing VOCs Using a Au/Pd-Modified RGO-SnO2 Gas Sensor Array
Source: Materials (Basel). 2026 Oct 30;19(21):4606. doi: 10.3390/ma19214606 (PMC13764613; doi:10.3390/ma19214606)
Supplement: Supplementary file 1 [file materials-19-04606-s001.zip › materials-4624875-supplementary.pdf]

Supplementary Materials

Intelligent Recognition of Sulfur-Containing VOCs Using an Au/Pd-Modified rGO–SnO_2 Gas Sensor Array

Heng Fu[1], Lin Shi[1], Yongfeng Kang[1], Yukang Liao[1] Hui Li[2]* Le Zheng[1]* and Jian Song [1]*

[1]School of Microelectronics, Shanghai University, Shanghai 200444, China;

[2] State Key Laboratory of High Performance Ceramics, Shanghai Institute of Ceramics, Chinese Academy of Sciences, Shanghai 200050, China

*Correspondence: lihui889@mail.sic.ac.cn (H. Li), lzheng@shu.edu.cn (L. Zheng), jsong@shu.edu.cn (J. Song)

S1. Preparation of the rGO-Modified Si/SiO_2 Substrate

First, an appropriate amount of reduced graphene oxide (rGO) was dispersed in deionized water to prepare an rGO dispersion with a concentration of 1 mg mL^{-1}. The dispersion was then subjected to ultrasonic treatment to promote sufficient exfoliation and dispersion of the rGO sheets. After sonication, the suspension was centrifuged to remove large agglomerates, and the stable supernatant was collected for subsequent use. Meanwhile, 1 mL of APTES was added to 49 mL of absolute ethanol and thoroughly mixed to prepare a 2% (v/v) APTES solution.

The cleaned Si/SiO_2 substrates were first pretreated by heating in an oven at 120 °C for 30 min. The substrates were then immersed in the prepared 2% APTES solution for 1 h for surface functionalization. After immersion, the substrates were removed and thoroughly rinsed with absolute ethanol to eliminate unbound APTES. The treated substrates were subsequently heated in an oven at 100 °C for 1 h to improve the stability of the surface functionalization layer.

After APTES treatment, 6 μL of the rGO dispersion was drop-cast onto the designated sensing area of each Si/SiO_2 substrate. The samples were then left undisturbed at room temperature for 2 h to allow sufficient deposition and formation of the rGO sensing layer. Finally, the samples were cleaned and dried, yielding rGO-modified Si/SiO_2 substrates for subsequent SnO_2 sensing-layer fabrication and device processing.

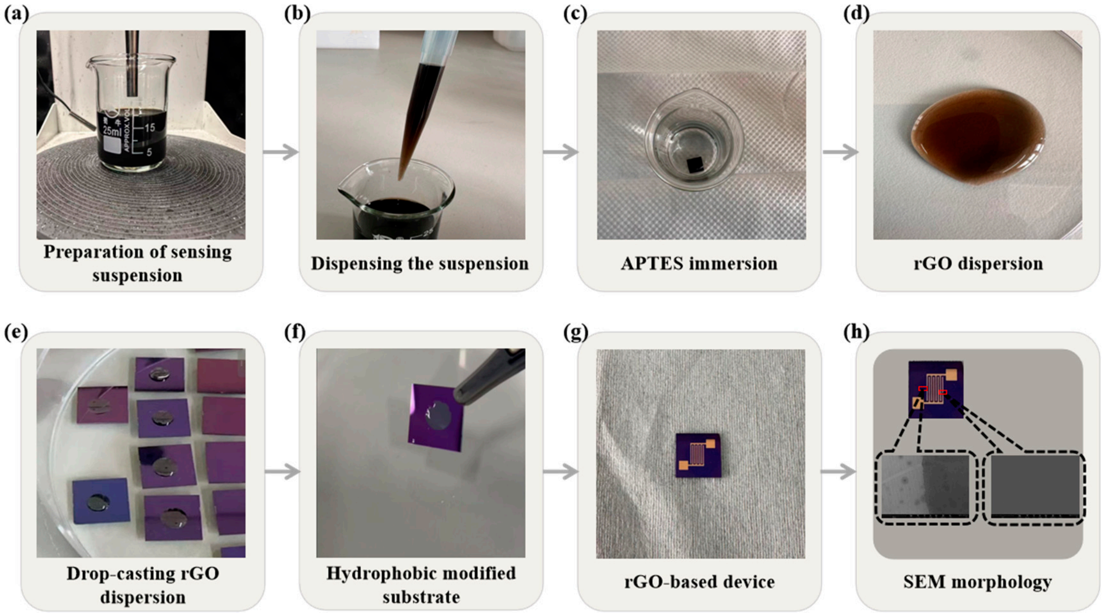

Figure S1. Photographs illustrating the fabrication process of the rGO-based gas sensor, including sensing suspension preparation, suspension dispensing, APTES treatment, rGO dispersion preparation, rGO drop-casting, substrate modification, device

fabrication, and SEM characterization.

S2. Preparation of SnO_2 Sol and Fabrication of Noble-Metal-Modified Gas Sensors

SnO_2 sol was prepared by dissolving 0.526 g of tin(IV) chloride pentahydrate ($SnCl_4 \cdot 5H_2O$) in 15 mL of ethylene glycol (EG) under magnetic stirring at 80 °C. The solution was stirred at 80 °C for 3 h, followed by heating at 120 °C for another 3 h to promote hydrolysis. Glycerol was then added at a volume ratio of 1:12 (glycerol to precursor solution, v/v), and stirring was continued to improve the film-forming properties of the sol. Next, 150 µL of the prepared sol was deposited onto a silicon substrate and spin-coated at 3000 rpm for 30 s. The coated substrate was heated on a hot plate at 100 °C for 10 min in air, followed by further heating at 400 °C for 1 h to form the SnO_2 sensing film.

For noble-metal modification, Au or Pd was deposited separately onto the sensing films by thermal evaporation at a rate of 0.5 Å/s, with a nominal thickness of 1 nm. The modified samples were subsequently annealed in an oven at 200 °C for 1 h to promote nanoparticle formation. The fabricated sensors were then used for gas-sensing measurements.

S3. Supplementary Characterization Results

Table S1. XPS peak parameters and elemental composition of the rGO–SnO_2–Pd sensor.

Element	Peak BE (eV)	FWHM (eV)	Atomic (%)
**Au 4f**	83.43	0.93	5.14
**C 1s**	283.84	1.57	33.66
**O 1s**	530.84	3.72	42.04
**Pd 3d**	334.73	1.85	13.21
**Si 2p**	101.02	0.24	3.07
**Sn 3d**	486.07	1.37	2.89

Table S2. XPS peak parameters and elemental composition of the rGO–SnO_2–Au sensor.

Element	Peak BE (eV)	FWHM (eV)	Atomic (%)
**Au 4f**	83.11	0.87	6.13
**C 1s**	283.91	1.64	41.33
**O 1s**	531.11	3.38	45.00
**Si 2p**	101.36	1.76	5.36
**Sn 3d**	486.03	1.39	2.18

Table S3. XPS peak parameters and elemental composition of the rGO–SnO_2 sensor.

Element	Peak BE (eV)	FWHM (eV)	Atomic (%)
**Au 4f**	84.26	0.94	5.83
**C 1s**	284.92	1.76	21.55
**O 1s**	532.04	2.77	49.98
**Si 2p**	102.99	2.00	13.08
**Sn 3d**	487.42	1.55	9.55

To further compare the surface compositions of the three sensing materials, the XPS peak parameters and elemental atomic percentages of the pristine rGO–SnO_2, Au/rGO–SnO_2, and Pd/rGO–SnO_2 sensors are summarized in Tables S1–S3. The pristine sensor exhibited C, O, Si, and Sn signals, with atomic percentages of 21.55%, 49.98%, 13.08%, and 9.55%, respectively. The Au-modified sensor showed an Au content of 6.13%, while the Pd-modified sensor exhibited a Pd content of 13.21%. An Au 4f signal was also detected in the pristine and Pd-modified sensors, which may originate from the Ti/Au interdigitated electrodes used in the device structure. These results provide quantitative information on the surface elemental compositions of the three sensors and support the presence of noble metals in the corresponding modified devices.

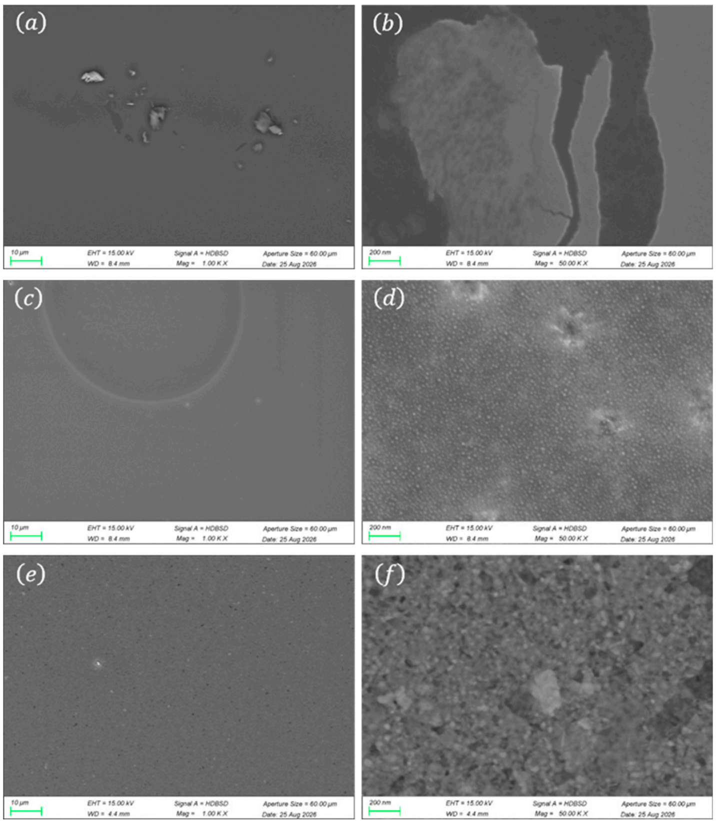

Figure S2. SEM images of the pristine and noble-metal-modified rGO–SnO_2 sensing films at different magnifications: (a,b) pristine rGO–SnO_2, (c,d) Au-modified rGO–SnO_2, and (e,f) Pd-modified rGO–SnO_2.

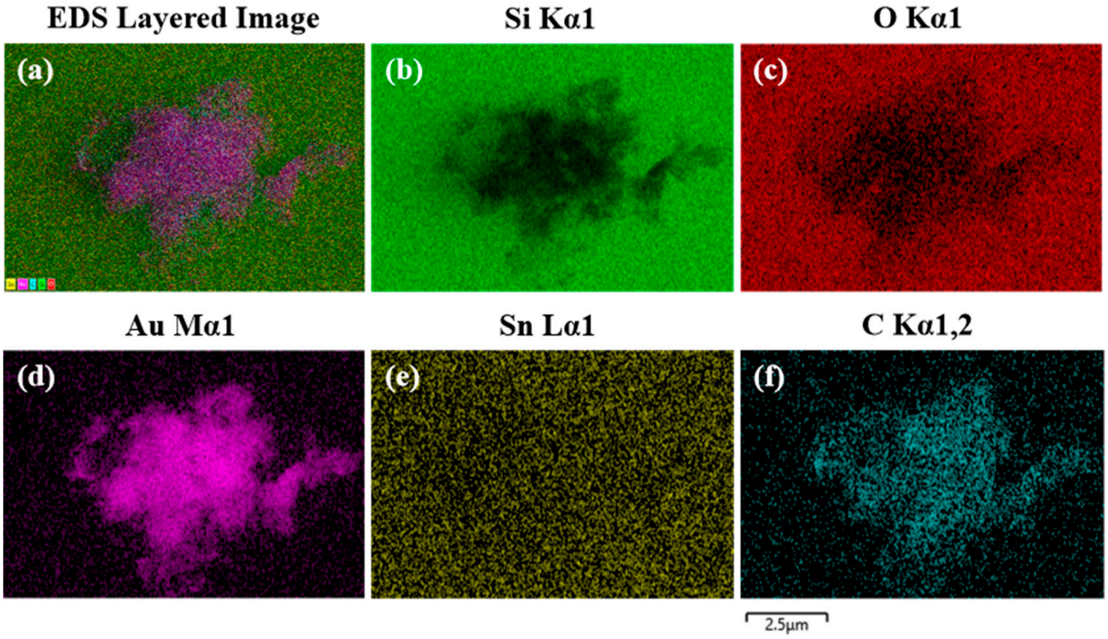

Figure S3. EDS elemental mapping images of the rGO–SnO_2–Au sensor: (a) elemental overlay and (b–f) individual elemental distribution maps of Si, O, Au, Sn and C, respectively.

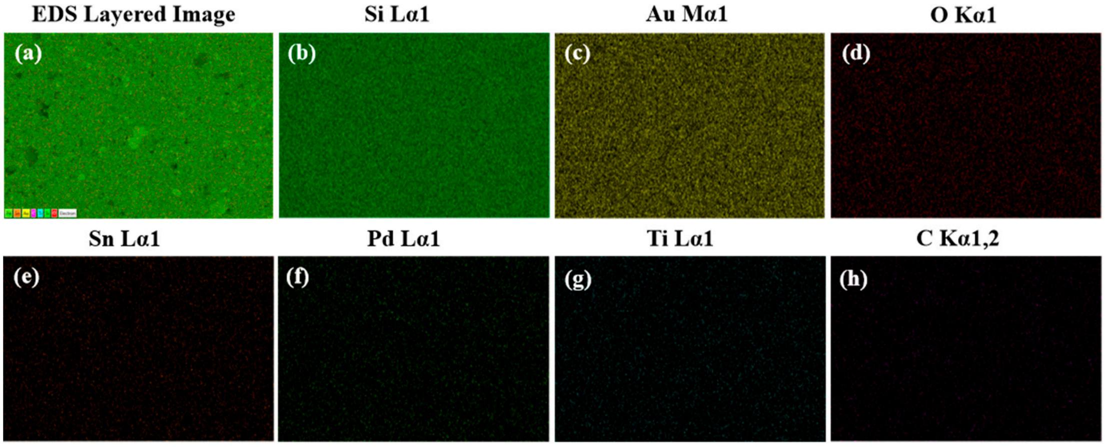

Figure S4. EDS elemental mapping images of the rGO–SnO_2–Pd sensor: (a) elemental overlay and (b–h) individual elemental distribution maps of Si, Au, O, Sn, Pd, Ti, and C, respectively.

S4. Supplementary Electrical Characterization and Experimental Conditions

S4.1. Supplementary Electrical Characterization

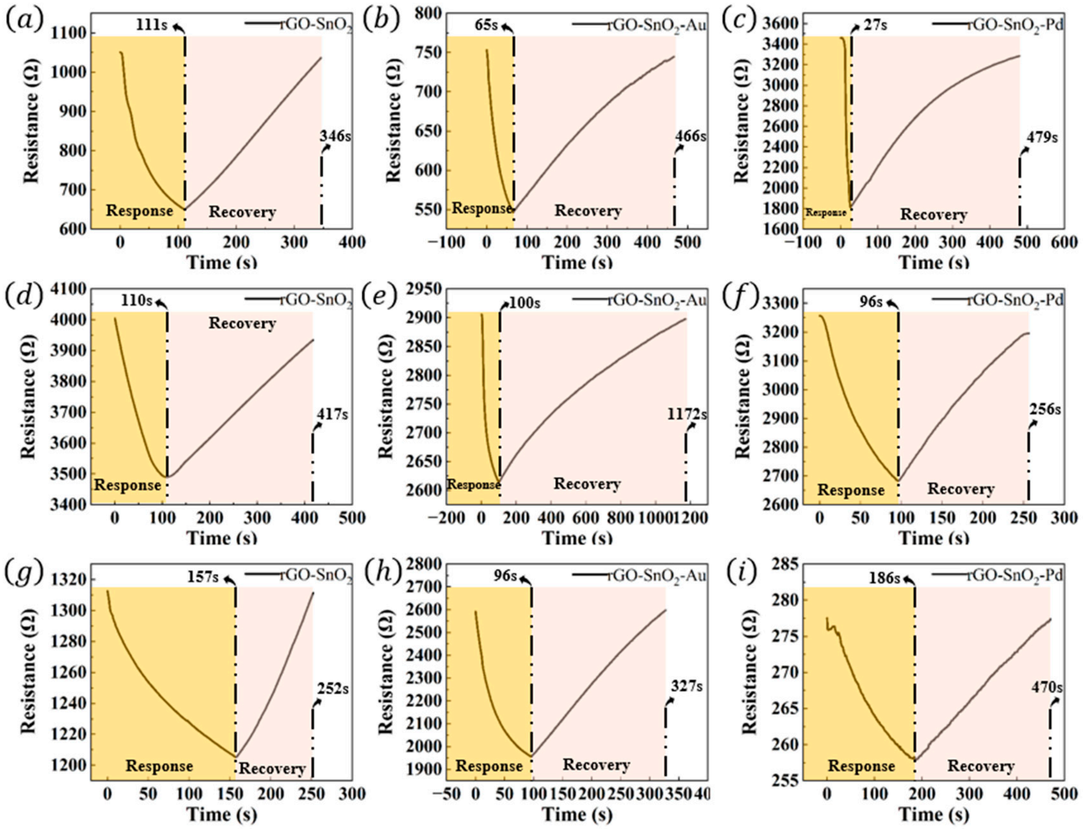

Figure S5. Response and recovery characteristics of the rGO-SnO_2-based sensor array toward sulfur-containing VOCs: (a–c) 1-BT, (d–f) DMS, and (g–i) AMS. The three columns correspond to rGO-SnO_2, rGO-SnO_2-Au, and rGO-SnO_2-Pd sensors, respectively.

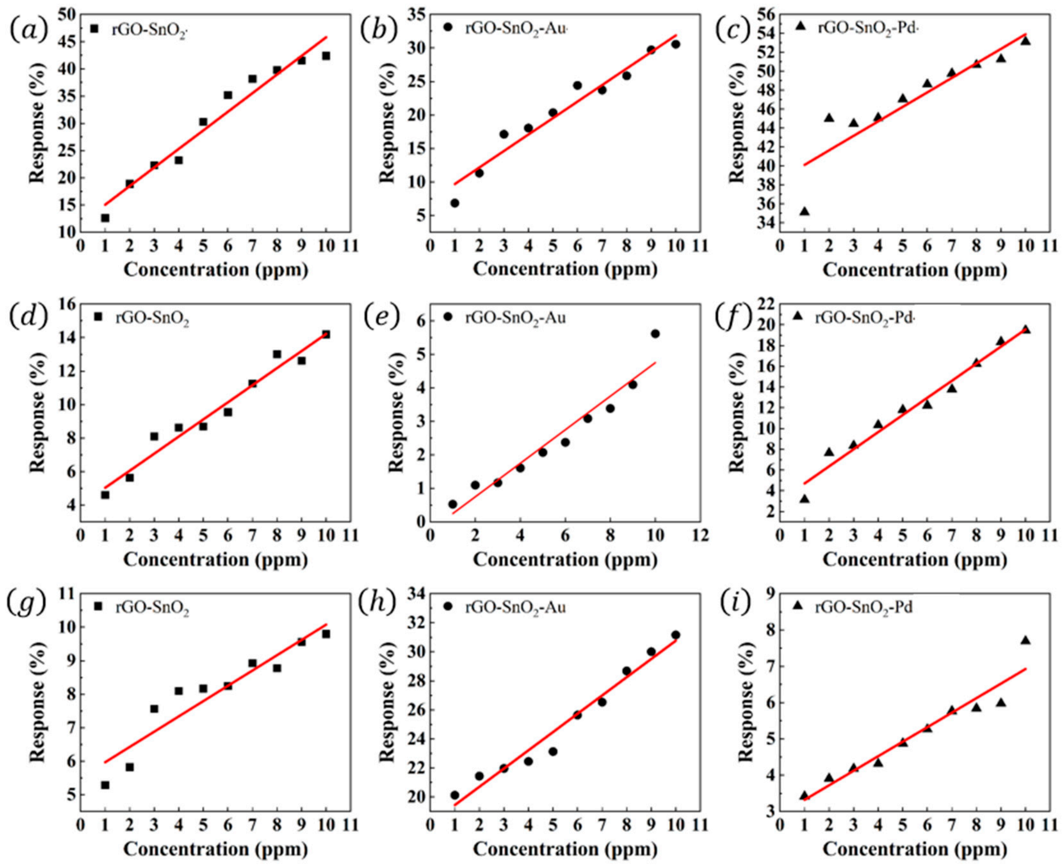

Figure S6. Calibration curves of the responses of rGO–SnO_2, rGO–SnO_2–Au, and rGO–SnO_2–Pd sensors toward different concentrations of butanethiol (first row), dimethyl sulfide (second row), and allyl methyl sulfide (third row).

S4.2. Gas-Sensing Conditions, Humidity Effects, and Practical Limitations

Gas-sensing measurements were carried out at room temperature in a sealed chamber. The volume of each liquid VOC was calculated from the chamber volume and the target gas concentration. The calculated amount was then placed on a heating plate to facilitate evaporation, while a fan was used to distribute the vapor throughout the chamber. As the actual vapor concentrations were not measured independently by gas chromatography–mass spectrometry (GC-MS) or other analytical methods, the concentrations reported here are nominal values.

The effect of humidity was examined using 10 ppm 1-butanethiol (1-BT), which produced relatively strong responses in the sensor array. All measurements were performed at room temperature. High humidity was achieved using a humidifier, while lower humidity levels were obtained by introducing dry air into the chamber. A humidity sensor connected to the humidifier was placed inside the chamber to monitor the relative humidity in real time. Gas exposure measurements were performed after the relative humidity inside the chamber had reached the target level and stabilized. The responses of the pristine rGO–SnO_2, Au-modified rGO–SnO_2, and Pd-modified rGO–SnO_2 sensors were measured at 10%, 30%, 50%, 70%, and 90% RH. The response was calculated as

$$Response = \frac{R_a}{R_g}$$

where (R_a) and (R_g) are the resistances measured in air and in the target gas, respectively, at the same humidity level.

Figure S7 shows the responses of the three sensors at different humidity levels. All three exhibited lower responses as the relative humidity increased. The response of the pristine rGO–SnO_2 sensor decreased gradually across the measured range. The Au-modified sensor showed a smaller change in its response ratio, while the Pd-modified sensor exhibited a more noticeable decrease, particularly between 10% and 30% RH. The reduced response at higher humidity may result from water molecules competing with 1-BT for adsorption sites on the sensing surface and affecting surface charge transfer. Despite this decrease, all three sensors retained measurable responses at 90% RH.

The concentrations used in this study were also compared with those reported in clinical breath measurements. Sukul et al. reported median breath concentrations of 14.8 ppbV for dimethyl sulfide (DMS), 4.30 ppbV for allyl methyl sulfide (AMS), and 1.21 ppbV for butanethiol in their discovery cohort. The lowest nominal concentration tested in this study was 100 ppb, approximately 6.8, 23.3, and 82.6 times the corresponding reported values, respectively. Thus, the concentrations investigated here remain higher than those typically found in the cited breath study.

Several limitations remain. The actual vapor concentrations were not independently verified, and losses caused by incomplete evaporation or adsorption onto the chamber walls cannot be ruled out. Humidity measurements were performed only for 1-BT, and the effects of humidity on DMS and AMS were not examined. Interference from other common breath constituents under mixed-gas conditions was also not assessed. Therefore, the present findings establish the sensing performance of the array under laboratory conditions but do not demonstrate its suitability for clinical osteoporosis screening. Further tests at lower concentrations, with verified vapor concentrations, mixed-gas exposure, and real breath samples will be needed to assess its potential for breath analysis.

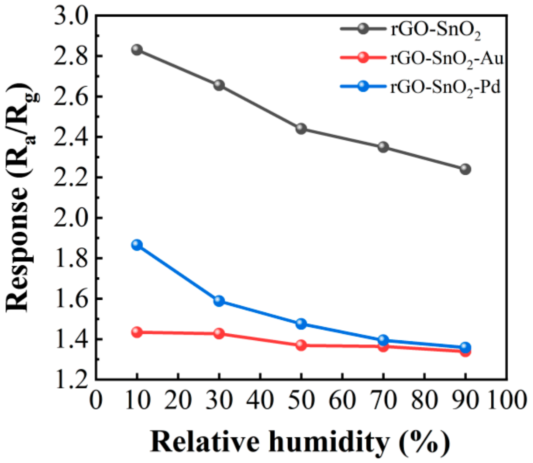

Figure S7. Effect of relative humidity on the sensing responses of pristine rGO–SnO_2, rGO-SnO_2-Au, and rGO–SnO_2-Pd sensors toward 10 ppm 1-butanethiol (1-BT).

S4.3. Repeatability and Stability Assessment

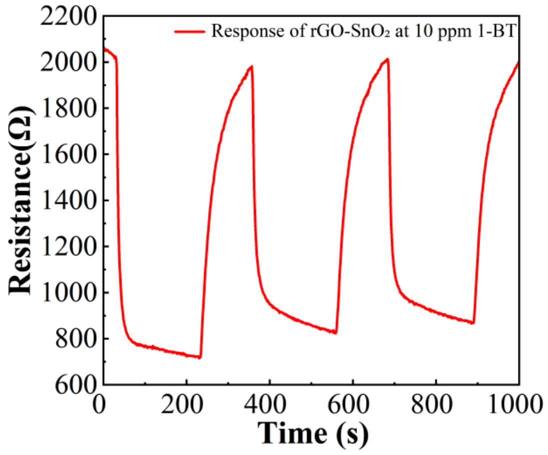

Figure S8. Repeated response–recovery cycles of the rGO–SnO_2 sensor after four months of storage under exposure to 10 ppm 1-BT.

To further evaluate the stability of the sensor, an rGO–SnO_2 sensor fabricated four months earlier was retested using 10 ppm 1-butanethiol (1-BT). As shown in Figure S8, the sensor exhibited repeated resistance changes during successive gas exposure cycles, indicating that it retained its sensing response after four months of storage. However, some baseline variation was observed between the cycles.

S5. Dynamic Gas-Sensing Measurement System

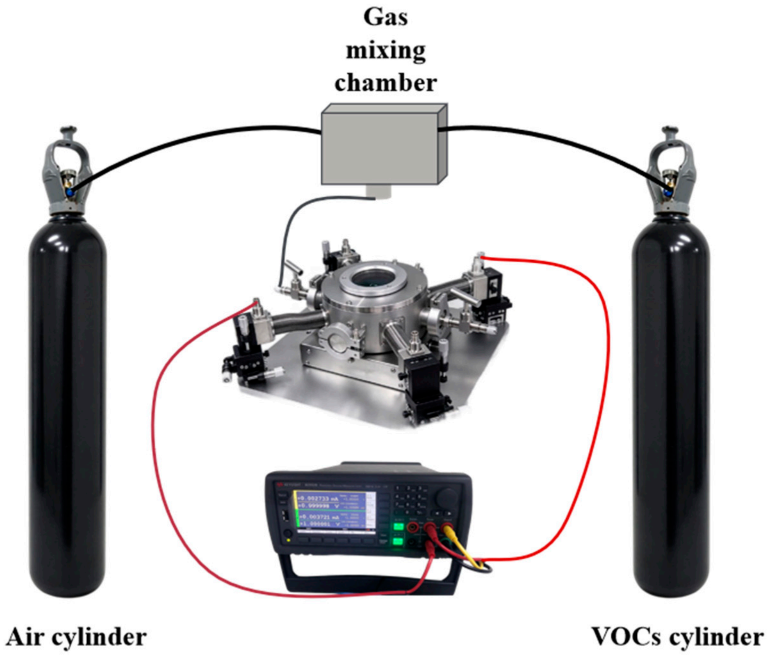

Figure S9. Dynamic Gas Sensing Test System

S6. Supplementary Details of the Hardware Circuit

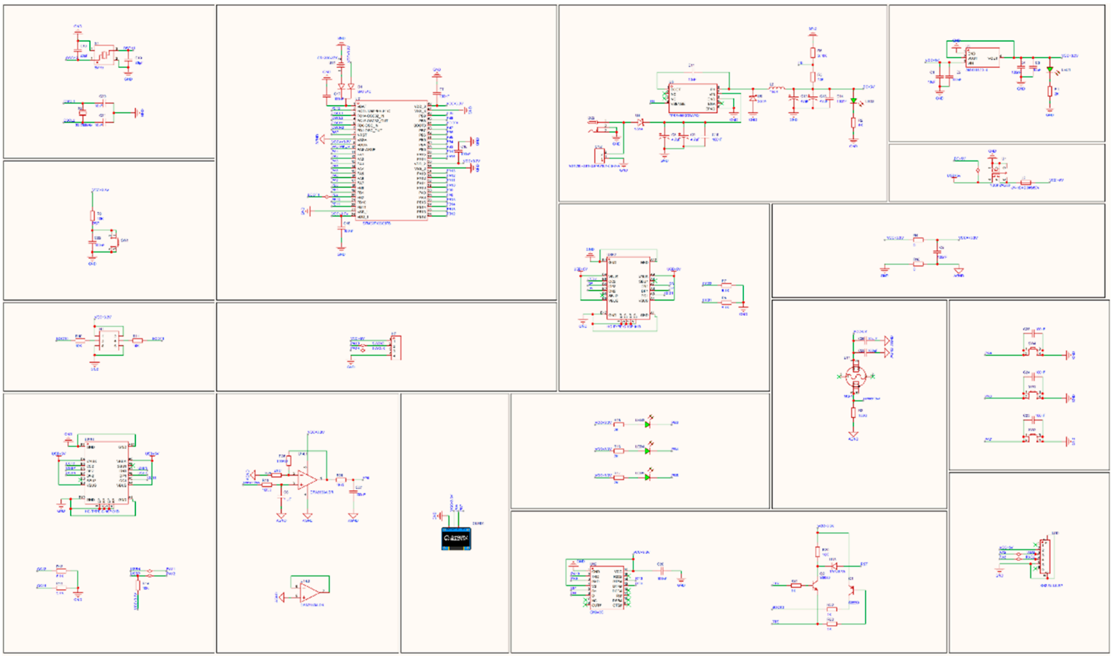

Figure S10. Schematic diagram of the hardware circuit.

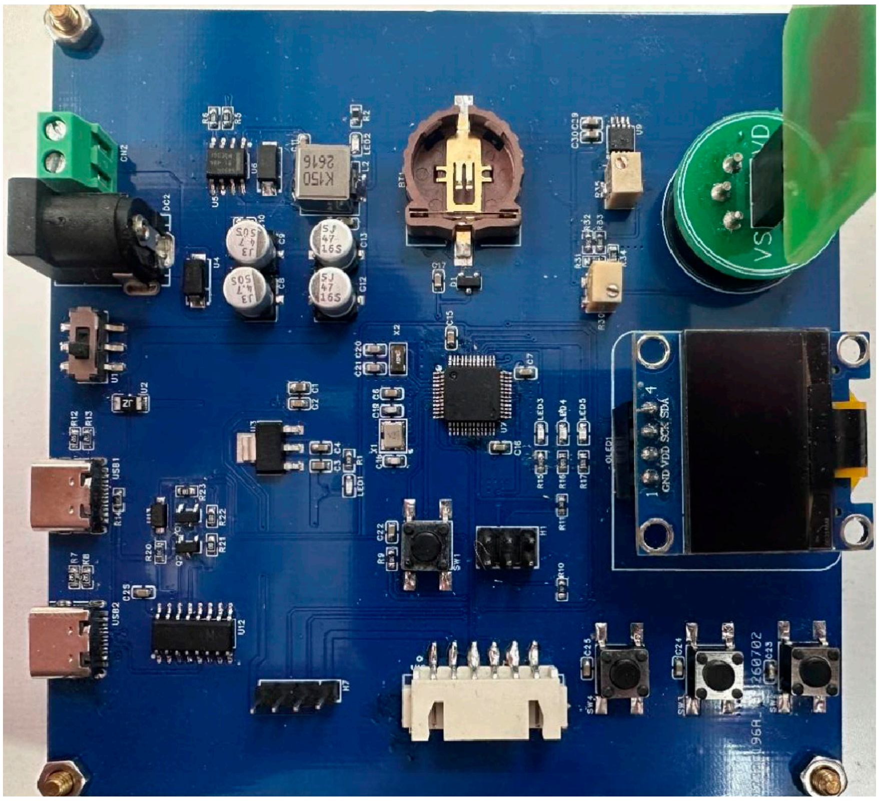

Figure S11. Photograph of the Fabricated Hardware Circuit

S7. Model Implementation and Experimental Validation

S7.1. Dataset Construction and Exposure-Run-Independent Validation

The dataset comprised nine gas recognition classes, covering three sensor configurations, three target gases, and four concentration levels. In total, 64 independent gas exposure runs were performed. Each run involved simultaneous measurements from 30 sensing devices under a specific gas type and concentration during a single exposure cycle. After excluding abnormal measurements, 27–30 valid response curves were obtained from each run, yielding 1,825 curves with 357 sampling points per curve.

The data were divided into training and test sets by exposure run rather than by individual curve. Each curve was linked to its original run using the gas type, concentration, and measurement cycle. All curves collected within the same run were kept in one subset, so no exposure runs were shared between training and testing. The resulting split was 80:20, and both subsets contained all 12 gas–concentration combinations. The training and test sets contained 51 and 13 independent exposure runs, respectively.

The model was trained three times using random seeds of 13, 7, and 4. With this run-based split, the average accuracy was 93.70% ± 0.59%, while macro-precision, macro-recall, and macro-F1-score were 93.98% ± 0.56%, 93.86% ± 0.63%, and 93.82% ± 0.58%, respectively. The accuracy was 1.72 percentage points lower than the 95.42% obtained with random curve-level splitting. Under the exposure-run-based split, the model maintained an average accuracy of 93.70%, suggesting that it retained good recognition performance on gas exposure runs not included in training.

Table S4. Classification performance of the proposed model under different data partitioning strategies.

Evaluation metric	Random curve-level split (%)	Exposure-run-based split (%)
**Accuracy**	95.42 ± 0.34	93.70 ± 0.59
**Macro-Precision**	95.46 ± 0.32	93.98 ± 0.56
**Macro-Recall**	95.45 ± 0.36	93.86 ± 0.63
**Macro-F1-score**	95.39 ± 0.35	93.82 ± 0.58

Note: Values are reported as mean ± standard deviation over three runs with random seeds of 13, 7, and 4. In the exposure-run-based split, all response curves from the same gas exposure run were assigned exclusively to either the training or test set.

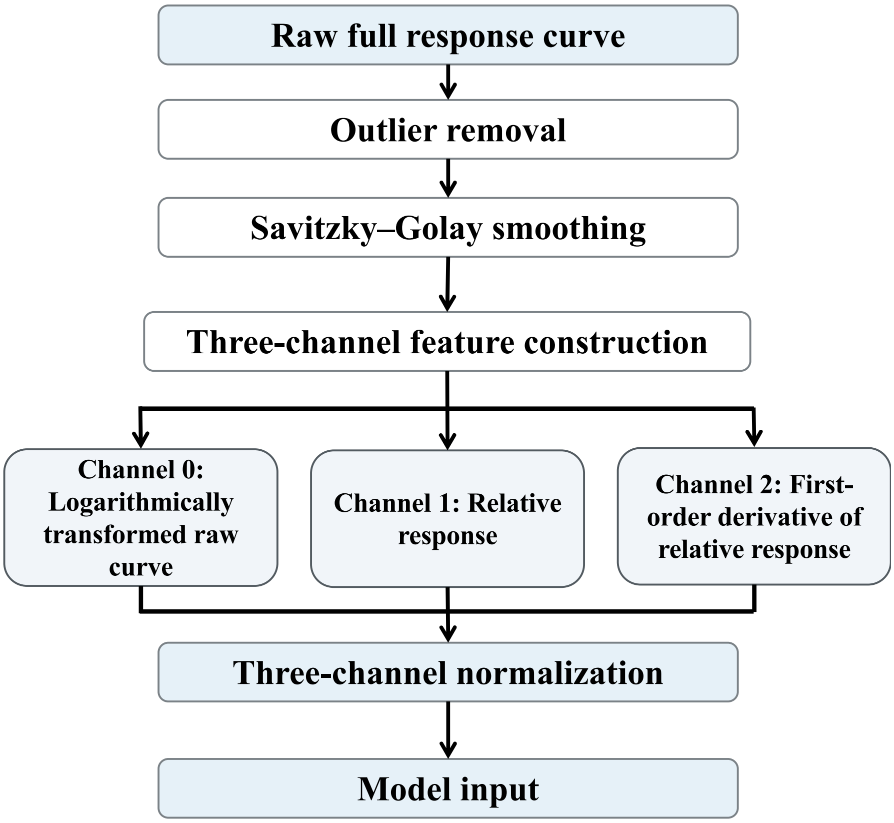

Figure S12. Workflow of sensor response preprocessing and three-channel feature construction.

S7.2. Baseline Comparisons and Ablation Studies

The three-branch multi-scale 1D-ResNet was compared with four commonly used classification methods: PCA–LDA, random forest (RF), radial basis function support vector machine (RBF-SVM), and a three-branch 1D-CNN. Both random sample splitting and exposure-run-based splitting were considered. Within each splitting strategy, all models used the same training and test sets. Classification performance was evaluated using accuracy, macro precision, macro recall, and macro F1-score, with the results reported as mean ± standard deviation.

Table S5 summarizes the results obtained with random sample splitting. The proposed model achieved an accuracy of 95.42% and a macro F1-score of 95.39%. Among the baseline models, the three-branch 1D-CNN performed best, reaching an accuracy of 92.86%, followed by RBF-SVM (86.54%), RF (85.35%), and PCA–LDA (64.56%). The accuracy of the proposed model was 2.56 percentage points higher than that of the 1D-CNN.

Table S5. Classification performance of the proposed model and baseline methods under random curve-level splitting.

Model	Accuracy (%)	Macro Precision (%)	Macro Recall (%)	Macro F1-score (%)
**PCA-LDA**	64.56 ± 0.00	68.41 ± 0.00	64.35 ± 0.00	64.38 ± 0.00
**Random Forest**	85.35 ± 0.34	85.34 ± 0.34	85.19 ± 0.32	85.03 ± 0.33
**RBF-SVM**	86.54 ± 0.00	87.16 ± 0.00	86.39 ± 0.00	86.29 ± 0.00
**1D-CNN**	92.86 ± 0.59	93.25 ± 0.51	92.85 ± 0.60	92.80 ± 0.58
**Proposed model**	95.42 ± 0.34	95.46 ± 0.32	95.45 ± 0.36	95.39 ± 0.35

A separate comparison was performed using exposure-run-based splitting to examine the model's performance on independent gas exposure runs. The training set comprised 1,460 samples from 51 exposure runs, while the test set contained 365 samples from 13 different runs. No exposure runs were shared between the two sets. As listed in Table S6, the proposed model achieved an accuracy of 93.70% and a macro F1-score of 93.82%. The corresponding accuracy of the three-branch 1D-CNN was 92.15%, giving a difference of 1.55 percentage points. The accuracy standard deviation was also lower for the proposed model (0.59%) than for the 1D-CNN (1.31%). Although the accuracy decreased slightly compared with random splitting, the proposed model remained the best-performing method among those evaluated.

Table S6. Classification results of the proposed model and baseline methods under exposure-run-based splitting.

Model	Accuracy (%)	Macro Precision (%)	Macro Recall (%)	Macro F1-score (%)
**PCA-LDA**	62.47 ± 0.00	66.66 ± 0.00	63.00 ± 0.00	63.25 ± 0.00
**Random Forest**	86.85 ± 0.39	87.26 ± 0.39	87.11 ± 0.41	86.93 ± 0.38
**RBF-SVM**	84.11 ± 0.00	84.67 ± 0.00	84.30 ± 0.00	84.17 ± 0.00
**1D-CNN**	92.15 ± 1.31	92.92 ± 1.08	92.21 ± 1.47	92.19 ± 1.41
**Proposed model**	93.70 ± 0.59	93.98 ± 0.56	93.86 ± 0.63	93.82 ± 0.58

The contribution of individual input channels and network components was examined through ablation experiments. Six variants were evaluated by separately removing the log-transformed raw signal channel, relative response channel, derivative channel, multi-scale convolution module, residual connections, and squeeze-and-excitation (SE) module. The remaining model settings and data partitions were kept unchanged for each comparison.

For random sample splitting, the ablation results are given in Table S7. Removing the log-transformed raw signal channel reduced accuracy from 95.42% to 90.29%, a

decrease of 5.13 percentage points. Accuracy also dropped to 92.31% when the derivative channel was removed. In contrast, excluding the relative response channel reduced accuracy by only 0.18 percentage points. The average accuracy remained at 95.42% without the multi-scale module and increased to 95.97% without residual connections. Removing the SE module resulted in a small decrease to 95.24%. Thus, the log-transformed raw signal and derivative channels had a greater effect on classification accuracy than the tested network modules under random splitting.

Table S7. Input-channel and network-module ablation results under random curve-level splitting.

Input configuration	Accuracy (%)	Macro Precision (%)	Macro Recall (%)	Macro F1-score (%)
**Without log-transformed raw signal**	90.29 ± 1.91	90.81 ± 1.72	90.50 ± 1.81	90.41 ± 1.84
**Without relative response**	95.24 ± 0.72	95.39 ± 0.74	95.32 ± 0.70	95.24 ± 0.73
**Without derivative**	92.31 ± 0.39	92.46 ± 0.38	92.34 ± 0.43	92.29 ± 0.42
**Without multi-scale module**	95.42 ± 0.26	95.53 ± 0.24	95.49 ± 0.30	95.41 ± 0.29
**Without residual connections**	95.97 ± 0.91	96.07 ± 0.90	96.01 ± 0.92	95.97 ± 0.90
**Without SE module**	95.24 ± 0.52	95.24 ± 0.48	95.23 ± 0.52	95.19 ± 0.50
**Full model**	95.42 ± 0.34	95.46 ± 0.32	95.45 ± 0.36	95.39 ± 0.35

Table S8 presents the ablation results obtained with exposure-run-based splitting. The largest accuracy reduction again occurred when the log-transformed raw signal channel was removed, with accuracy falling from 93.70% to 88.13%. Removing residual connections reduced accuracy to 92.69%, a decrease of 1.01 percentage points. The other modifications had smaller effects. Accuracy decreased to 93.52% without the relative response channel, 93.61% without the derivative channel, and 93.42% without the multi-scale module. Excluding the SE module gave an accuracy of 93.58%, only 0.12 percentage points below the full model. The effect of residual connections therefore differed between the two splitting strategies, while the log-transformed raw signal channel remained important in both cases.

Table S8. Input-channel and network-module ablation results under exposure-run-based splitting.

Input configuration	Accuracy (%)	Macro Precision (%)	Macro Recall (%)	Macro F1-score (%)
**Without log-transformed raw signal**	88.13 ± 0.93	88.59 ± 0.86	88.56 ± 0.95	88.30 ± 0.90
**Without relative response**	93.52 ± 1.01	93.80 ± 1.02	93.69 ± 0.93	93.66 ± 1.00
**Without derivative**	93.61 ± 0.26	93.81 ± 0.31	93.76 ± 0.26	93.69 ± 0.27
**Without multi-scale module**	93.42 ± 1.03	93.65 ± 1.03	93.61 ± 0.98	93.57 ± 1.01
**Without residual connections**	92.69 ± 0.47	93.09 ± 0.43	92.97 ± 0.48	92.93 ± 0.45
**Without SE module**	93.58 ± 0.56	93.64 ± 0.61	93.79 ± 0.59	93.66 ± 0.58
**Full model**	93.70 ± 0.59	93.98 ± 0.56	93.86 ± 0.63	93.82 ± 0.58

The proposed model achieved higher average classification accuracy than the selected baseline methods under both data splitting strategies. Among the components examined, the log-transformed raw signal channel showed the clearest contribution to performance. Residual connections improved accuracy under exposure-run-based splitting but not under random splitting. The improvements associated with the multi-scale and SE modules were comparatively small. These observations suggest that the performance gain of the complete model cannot be attributed equally to all its components, and that some architectural simplifications may be possible without substantially affecting classification accuracy.

S8. Comparison with Previous Studies

Table S9. Comparison of the proposed gas sensing system with previously reported studies.

Study	Sensing materials	Target gases/samples	Recognition method	Hardware / system	Main contribution
**Bhangare et al. (2022)[16]**	Pt/Pd–SnO_2/rGO	H_2, H_2S, NO_2	PCA, LDA, HCA	Chemiresistive E-nose	Noble-metal sensitization and gas discrimination
**Acharyya et al. (2023)[36]**	Ag/Au/Pd/Pt–SnO_2	Multiple VOCs	DNN	Interdigitated sensor array	VOC classification and quantification
**Moon et al. (2024)[37]**	rGO, rGO/ZnO, rGO/TiO_2, rGO/SnO_2	NO_2, NO, SO_2	ML	Flexible sensor array	Room-temperature gas discrimination
**Chen et al. (2024)[38]**	Graphene-based materials	Breath VOCs / breath samples	MLP	Mobile E-nose with ADC	Breath analysis and hardware integration
**Sun et al. (2024)[35]**	Electronic-nose dataset	Gas classification dataset	SnapE-ResNet	Open sampling system	Drift compensation and ensemble recognition
**Huang et al. (2021)[39]**	Commercial MOS sensors	Wine and oil samples	SVM	Portable STM32-based E-nose	Integrated acquisition and classification
**This work**	rGO–SnO_2, Au/rGO–SnO_2, Pd/rGO–SnO_2	DMS, AMS, 1-BT	Multibranch multiscale ResNet	Dedicated signal acquisition circuit	Differentiated sulfur-VOC sensing and dynamic recognition

Previous studies have explored noble-metal-modified metal oxide sensors and machine-learning methods for gas recognition. Bhangare et al. [15] developed a Pt/Pd-modified SnO_2/rGO electronic nose to distinguish H_2, H_2S, and NO_2, while Sun et al. [34] applied a ResNet-based method to gas classification. In this study, pristine, Au-modified, and Pd-modified rGO–SnO_2 sensors were used to obtain different responses toward three sulfur-containing VOCs. A multibranch, multiscale ResNet model was then employed to classify the gas response signals, and a dedicated signal acquisition and processing circuit was designed for the sensor array. The main differences between

this study and previous work are summarized in Table S9.

Table S10. Comparison of the proposed sensors with commercially available and previously reported gas sensors.

Sensor composition	Detection medium	Detection method	Response	Year
**Figaro TGS2603 (Commercial MOS sensor)**	Methyl mercaptan, sulfurous odor gases	Chemiresistive	R_s/R_{air}<0.5 (10 ppm ethanol)	2013[40]
**Au/ZnO with sepiolite filter**	DMS(1 ppm,90% RH)	Chemiresistive	S=11.4	2024[41]
**Pt/SnO_2 nanosheets**	DMS (1 ppm)	Chemiresistive	R_a/R_g=4.84	2018[42]
**rGO–SnO_2, Au/rGO–SnO_2, Pd/rGO–SnO_2**	1-BT/AMS/DMS	Chemiresistive	R_a/R_g=2.84 (rGO–SnO_2 in BT)	2026

Note: The reported response values were obtained using different response definitions, target gases, and experimental conditions and are therefore not directly comparable. The commercial sensor specifications refer to the manufacturer's standard test conditions.

Algorithm S1. Three-Branch Multi-Scale 1D-ResNet for Sulfur-Containing VOC Classification

```
Input:
    D_train = {(X_i, y_i)}_(i=1)^(N_train)
    D_test  = {(X_j, y_j)}_(j=1)^(N_test)
    X ∈ R^357

    D1 = rGO/SnO2
    D2 = Au-rGO/SnO2
    D3 = Pd-rGO/SnO2

    BT  = 1-Butanethiol
    AMS = Allyl methyl sulfide
    DMS = Dimethyl sulfide

    L1 = D1 | BT
    L2 = D1 | AMS
    L3 = D1 | DMS
    L4 = D2 | BT
    L5 = D2 | AMS
    L6 = D2 | DMS
    L7 = D3 | BT
    L8 = D3 | AMS
    L9 = D3 | DMS

    Random seeds S = {13, 7, 4}

Output:
    Predicted class y_hat ∈ {L1, ..., L9}
    Accuracy, Macro Precision, Macro Recall, Macro F1-score
    Mean confusion matrix

1:  Load D_train and D_test

2:  for each response curve X do
3:      Interpolate missing values along the time axis
4:      X_s ← SavitzkyGolay(X, window_length = 11, polyorder =
2)
5:      B ← median(X_s[1:36])
6:      X_log ← log(max(X_s, 10^-8))
```

```
    X_rel ← (X_s - B) / max(|B|, 10^-8)
    X_rel ← clip(X_rel, -20, 20)
    X_der ← gradient(X_rel)
    Z ← Stack(X_log, X_rel, X_der)
end for

Compute channel-wise μ_c and σ_c using D_train only
for each channel c do
    Z'_train,c ← (Z_train,c - μ_c) / σ_c
    Z'_test,c  ← (Z_test,c  - μ_c) / σ_c
end for

for each random seed s ∈ S do
    Set random seed s for Python, NumPy, and PyTorch
    Initialize ThreeBranchMultiScale1DResNet

    for each branch b ∈ {X_log, X_rel, X_der} do
        H_b ← Conv1D(X_b, kernel_size = 9, out_channels = 24)
        H_b ← BatchNorm(H_b)
        H_b ← ReLU(H_b)
        H_b ← MaxPool1D(H_b, kernel_size = 2)

        H_b ← MSResBlock(H_b, C_in = 24, C_out = 48)
        H_b ← MaxPool1D(H_b, kernel_size = 2)

        H_b ← MSResBlock(H_b, C_in = 48, C_out = 72)
        H_b ← MaxPool1D(H_b, kernel_size = 2)

        H_b ← MSResBlock(H_b, C_in = 72, C_out = 72)
        H_b ← AdaptiveAveragePooling1D(H_b, output_length = 8)
        H_b ← Flatten(H_b)
    end for

    H ← Concat(H_log, H_rel, H_der)
    H ← FullyConnected(H, input_dim = 72 × 8 × 3, output_dim = 192)
    H ← ReLU(H)
    H ← Dropout(H, p = 0.35)
    logits ← FullyConnected(H, input_dim = 192, output_dim = 9)
```

```
    Initialize CrossEntropyLoss
    Initialize AdamW:
       learning_rate = 1 × 10^-3
       weight_decay = 1 × 10^-4
    Initialize CosineAnnealingLR:
       T_max = 100
       eta_min = 1 × 10^-5

    for epoch = 1 to 100 do
       Set model to training mode
       for each mini-batch (Z_batch, y_batch), batch_size = 64 do
          logits_batch ← Model(Z_batch)
          L_CE ← CrossEntropyLoss(logits_batch, y_batch)
          Clear optimizer gradients
          Backpropagate L_CE
          Update network parameters using AdamW
       end for
       Update learning rate using cosine annealing
    end for

    Set model to evaluation mode
    for each test batch Z_test,batch do
       logits_test ← Model(Z_test,batch)
       y_hat ← argmax(logits_test)
    end for

    Compute Accuracy(s)
    Compute Macro Precision(s)
    Compute Macro Recall(s)
    Compute Macro F1-score(s)
    Compute confusion matrix CM(s)
end for

Accuracy_mean  ← mean_s[Accuracy(s)]
Accuracy_std   ← std_s[Accuracy(s)]
Precision_mean ← mean_s[Macro Precision(s)]
Precision_std  ← std_s[Macro Precision(s)]
Recall_mean    ← mean_s[Macro Recall(s)]
Recall_std     ← std_s[Macro Recall(s)]
F1_mean        ← mean_s[Macro F1-score(s)]
F1_std         ← std_s[Macro F1-score(s)]
CM_mean        ← mean_s[CM(s)]
```

```
72: Return:
       Accuracy_mean ± Accuracy_std
       Precision_mean ± Precision_std
       Recall_mean ± Recall_std
       F1_mean ± F1_std
       CM_mean
```

Comments

Comment 1:
D1, D2, and D3 denote rGO/SnO_2, Au-rGO/SnO_2, and Pd-rGO/SnO_2, respectively.

Comment 2:
BT, AMS, and DMS denote 1-butanethiol, allyl methyl sulfide, and dimethyl sulfide.

Comment 3:
Each response curve contains 357 sampling points.

Comment 4:
The baseline B is calculated as the median of the first 36 sampling points.

Comment 5:
Three input representations are constructed:
X_log = log-transformed original response curve;
X_rel = relative response curve;
X_der = first-order derivative of X_rel.

Comment 6:
Only D_train is used to compute μ_c and σ_c.
The same normalization parameters are applied to D_test.

Comment 7:
Each branch shares the same feature-extraction structure.

Comment 8:
Each multi-scale residual block uses parallel Conv1D kernels of 3, 7, and 15.

Comment 9:
The channel dimensions are 24 → 48 → 72 → 72.

Comment 10:
SE channel attention is embedded in each multi-scale residual block.

Comment 11:
The three branch features H_log, H_rel, and H_der are concatenated before classification.

Comment 12:
The classifier uses 192 hidden units, ReLU, Dropout p = 0.35, and a 9-class output layer.

Comment 13:
Training uses 100 epochs and batch size 64.

Comment 14:
AdamW uses learning rate 1 × 10^-3 and weight decay 1 × 10^-4.

Comment 15:
Cosine annealing is used with eta_min = 1 × 10^-5.

Comment 16:
Three independent runs use random seeds 13, 7, and 4.

Comment 17:
Final metrics are reported as mean ± standard deviation across the three runs.

Comment 18:
CM_mean is the average confusion matrix across the three random seeds.

Algorithm S2. Multi-Scale Residual Block

```
Function MSResBlock(H_in, C_in, C_out):

1:  C_branch ← C_out / 3

2:  H_3  ← Conv1D(H_in, kernel_size = 3,  out_channels =
C_branch)
3:  H_7  ← Conv1D(H_in, kernel_size = 7,  out_channels =
C_branch)
4:  H_15 ← Conv1D(H_in, kernel_size = 15, out_channels =
C_branch)

5:  H_MS ← Concat(H_3, H_7, H_15)
6:  H_MS ← BatchNorm(H_MS)

7:  z ← GlobalAveragePooling1D(H_MS)
8:  z ← Conv1D(z, kernel_size = 1, out_channels = max(C_out /
8, 8))
9:  z ← ReLU(z)
10: a ← Conv1D(z, kernel_size = 1, out_channels = C_out)
11: a ← Sigmoid(a)
12: H_SE ← H_MS ⊙ a

13: if C_in = C_out then
14:     H_shortcut ← H_in
15: else
16:     H_shortcut ← Conv1D(H_in, kernel_size = 1, out_channels
= C_out)
17:     H_shortcut ← BatchNorm(H_shortcut)
18: end if

19: H_out ← ReLU(H_SE + H_shortcut)
20: return H_out
```

Comments

Comment 1:
MSResBlock denotes the multi-scale residual block.

Comment 2:
Kernel sizes 3, 7, and 15 capture short-, medium-, and long-range temporal patterns.

Comment 3:
The three convolution outputs are concatenated along the channel dimension.

Comment 4:
Batch normalization is applied after multi-scale feature concatenation.

Comment 5:
The SE module performs channel-wise feature recalibration.

Comment 6:
The SE reduction ratio is 8.

Comment 7:
When $C_{in} \neq C_{out}$, a 1×1 convolution aligns the shortcut channel dimension.

Comment 8:
The final residual output is obtained by $H_{SE} + H_{shortcut}$ followed by ReLU.

Symbol Definitions

X_i : Original dynamic response curve of the i-th sample.
X_s : Smoothed response curve after Savitzky–Golay filtering.
B : Baseline value calculated from the first 36 sampling points.
X_log : Log-transformed original response curve.
X_rel : Relative response curve.
X_der : First-order derivative of X_rel.
Z : Three-branch input representation [X_log, X_rel, X_der].

D1 : rGO/SnO_2 device.
D2 : Au-rGO/SnO_2 device.
D3 : Pd-rGO/SnO_2 device.

BT : 1-Butanethiol.
AMS : Allyl methyl sulfide.
DMS : Dimethyl sulfide.

L1-L9 : Nine device-gas combination classes.

H_log : Deep feature extracted from X_log.
H_rel : Deep feature extracted from X_rel.
H_der : Deep feature extracted from X_der.
H : Fused feature after concatenating H_log, H_rel, and H_der.

C_in : Number of input channels.
C_out : Number of output channels.

CM(s) : Confusion matrix under random seed s.
CM_mean : Mean confusion matrix across three random seeds.

y : Ground-truth class label.
y_hat : Predicted class label.
L_CE : Cross-entropy loss.
μ_c : Mean of channel c computed from D_train.
σ_c : Standard deviation of channel c computed from D_train.

References

[16] Bhangare, B.; Sinju, K.R.; Ramgir, N.S.; Gosavi, S.; Debnath, A.K. Noble metal sensitized SnO_2/RGO nanohybrids as chemiresistive E-nose for H_2, H_2S and NO_2 detection. Mater. Sci. Semicond. Process. 2022, 147, 106706.

[35] Sun, B.; Gan, W.; Ma, R.; Feng, P.; Chu, J. SnapE-ResNet: A novel electronic nose classification algorithm for gas data collected by open sampling systems. Sens. Actuators A Phys. 2024, 379, 115978.

[36] Acharyya, S.; Bhowmick, P.K.; Guha, P.K. Selective identification and quantification of VOCs using metal nanoparticles decorated SnO_2 hollow-spheres based sensor array and machine learning. J. Alloys Compd. 2023, 968, 171891.

[37] Moon, D.-B.; Bag, A.; Chouhdry, H.H.; Hong, S.J.; Lee, N.-E. Selective identification of hazardous gases using flexible, room-temperature operable sensor array based on reduced graphene oxide and metal oxide nanoparticles via machine learning. ACS Sens. 2024, 9, 6071–6081.

[38] Chen, Q.; Guo, X.; Jiang, Y.; Liu, X.; Xu, S.; Huang, X.; Chen, Y.; Ye, X.; Pan, A.; Dong, Y.; He, Z.; Wu, J. A mobile E-nose prototype for online breath analysis. Adv. Sens. Res. 2024, 3, 2300018.

[39] Huang, Y.; Doh, I.-J.; Bae, E. Design and validation of a portable machine learning-based electronic nose. Sensors 2021, 21, 3923.

[40] Figaro Engineering Inc. TGS2603: Product Information. Available online: https://www.figaro.co.jp/en/product/docs/tgs2603_product_information_rev02.pdf (accessed on 23 October 2026).

[41] Suchorska-Woźniak, P.; Teterycz, H. ZnO hexagonal nano- and microplates modified with nanomaterials as a gas-sensitive material for DMS detection—extended studies. Sensors 2024, 24, 5690.

[42] Jang, J.-S.; Lim, Y.-W.; Kim, D.-H.; Lee, D.; Koo, W.-T.; Lee, H.; Bae, B.-S.; Kim, I.-D. Glass-fabric reinforced Ag nanowire/siloxane composite heater substrate: Sub-10 nm metal@metal oxide nanosheet for sensitive flexible sensing platform. Small 2018, 14, 1802260.
